# Supplementary material for: Proteome-wide prediction of targets for aspirin: new insight into the molecular mechanism of aspirin
Source: PeerJ. 2016 Mar 10;4:e1791. doi: 10.7717/peerj.1791 (PMC4793309; doi:10.7717/peerj.1791)
Supplement: Table S1 — Of these proteins, 23 proteins were identified as putative targets of aspirin after molecular docking and binding free energy calculation. [file peerj-04-1791-s001.docx]

**Table S1. Detailed information for all 79 proteins which have similar binding sites of aspirin (BSAs).** Of these proteins, **23** proteins were identified as putative targets of aspirin after molecular docking and binding free energy calculation.

| **GENE SYMBOL** | **Uniprot Accession** | **Protein names** | **Docking energy (kcal/mol)** | **Number of**  **Docking pose** | **ΔG^PB^_binding_**  **(kcal/mol)** |
| --- | --- | --- | --- | --- | --- |
| EXOSC3 | Q9NQT5 | Exosome complex component RRP40 | -14.5 | 10 | -33.0 |
| MAPK12 | P53778 | Mitogen-activated protein kinase 12 | -14.0 | 10 | -28.6 |
| ITGAL | O43746 | Integrin alpha-L | 65.2 | 10 | -28.0 |
| PTGS1 | P23219 | Prostaglandin G/H synthase 1 | -27.0 | 10 | -27.6 |
| PLA2G10 | O15496 | Group X Secretory Phospholipase A2 | -27.0 | 10 | -25.7 |
| FBP1 | P09467 | Fructose-1,6-bisphosphatase 1 | -17.4 | 10 | -25.2 |
| CUL4B | Q13620 | Cullin-4B | -15.5 | 10 | -23.0 |
| PTGS2 | [P35354](http://www.uniprot.org/uniprot/P35354) | Prostaglandin G/H synthase 2 | -26.0 | 10 | -20.2 |
| MMP12 | B2R9X8 | Macrophage metalloelastase | -22.5 | 10 | -18.6 |
| CDK13 | Q14004 | Cyclin-dependent kinase 13 | 315.7 | 10 | -18.4 |
| TNFAIP6 | P98066 | Tumor necrosis factor-inducible protein TSG-6 | -8.3 | 2 | -16. 8 |
| PLA2G3 | Q9NZ20 | Group 3 secretory phospholipase A2 | -24.2 | 10 | -14. 7 |
| HLA-A | O19619 | MHC class I antigen | 325.5 | 9 | -12.1 |
| MOCS3 | O95396 | Adenylyltransferase and sulfurtransferase MOCS3 | -18.7 | 10 | -12.0 |
| AIDA | Q96BJ3 | Axin interactor, dorsalization-associated protein | -24.8 | 10 | -11.1 |
| RAC1 | P63000 | Ras-related C3 botulinum toxin substrate 1 isoform Rac1b | -20.2 | 10 | -11.0 |
| PLA2G5 | P39877 | Calcium-dependent phospholipase A2 | -24.2 | 10 | -10.8 |
| PLA2G1B | P04054 | Phospholipase A2 | -23.1 | 10 | -10.6 |
| PLA2G2A | P14555 | Phospholipase A2, membrane associated | -29.2 | 10 | -10.6 |
| TNFSF14 | O43557 | Tumor necrosis factor ligand superfamily member 14 | -17.2 | 10 | -10.3 |
| CHIT1 | Q13231 | Chitotriosidase-1 | -24.0 | 10 | -10.0 |
| EGFLAM | Q63HQ2 | Pikachurin | -21.2 | 10 | -9.2 |
| PLA2G2D | Q9UNK4 | Group IID secretory phospholipase A2 | -28.7 | 10 | -6.0 |
| MICAL2 | O94851 | Protein-methionine sulfoxide oxidase MICAL2 | -26.3 | 10 | -0.7 |
| PLA2G2F | Q9BZM2 | Group IIF secretory phospholipase A2 | -24.8 | 10 | 6.8 |
| LPO | P22079 | Lactoperoxidase | -18.3 | 10 | 12.1 |
| SERPING1 | P05155 | Plasma protease C1 inhibitor | Failure | 0 | None |
| RNF125 | Q96EQ8 | E3 ubiquitin-protein ligase RNF125 | Failure | 0 | None |
| TRIM7 | Q9C029 | Tripartite motif-containing protein 7 | Failure | 0 | None |
| NCEH1 | Q6PIU2 | Neutral cholesterol ester hydrolase 1 | Failure | 0 | None |
| LDLR | B4DJZ8 | Low-density lipoprotein receptor | Failure | 0 | None |
| SKP1 | D3DQ97 | S-phase kinase-associated protein 1A | Failure | 0 | None |
| NFATC2 | Q13468 | Nuclear factor of activated T-cells, cytoplasmic 2 | Failure | 0 | None |
| GAA | P10253 | Lysosomal alpha-glucosidase | Failure | 0 | None |
| VRL2 | Q9HBA0 | Transient receptor potential cation channel subfamily V member 4 | Failure | 0 | None |
| PVRL2 | Q92692 | Poliovirus receptor-related protein 2 | Failure | 0 | None |
| PIK3CA | P42336 | Phosphatidylinositol-4,5-bisphosphate 3-kinase catalytic sub | Failure | 0 | None |
| NQO2 | B2R492 | Ribosyldihydronicotinamide dehydrogenase [quinone] | Failure | 0 | None |
| CPA2 | A4D1M4 | Carboxypeptidase A2 | Failure | 0 | None |
| CLK2 | P49760 | Dual specificity protein kinase CLK2 | Failure | 0 | None |
| CCNA2 | P20248 | Cyclin-A2 | Failure | 0 | None |
| ATPBD4 | Q7L8W6 | ATP-binding domain-containing protein 4 | Failure | 0 | None |
| ARHGAP5 | A1L375 | Rho GTPase activating protein 5 variant | Failure | 0 | None |
| ADAM17 | P78536 | Disintegrin and metalloproteinase domain-containing protein 17 | Failure | 0 | None |
| ABCB11 | O95342 | Bile salt export pump | Failure | 0 | None |
| LEP | O15158 | Leptin | Failure | 0 | None |
| HS3ST2 | Q9Y278 | Heparan sulfate glucosamine 3-O-sulfotransferase 2 | Failure | 0 | None |
| CTSF | B2R964 | Cathepsin F | Failure | 0 | None |
| CDKL2 | B2R695 | Cyclin-dependent kinase-like 2 | Failure | 0 | None |
| TLE3 | Q04726 | Transducin-like enhancer protein 3 | Failure | 0 | None |
| PLXNA2 | O75051 | Plexin-A2 | Failure | 0 | None |
| IGDCC4 | Q8TDY8 | Immunoglobulin superfamily DCC subclass member 4 | Failure | 0 | None |
| HEPH | Q9BQS7 | Hephaestin | Failure | 0 | None |
| FCAR | P24071 | Immunoglobulin alpha Fc receptor | Failure | 0 | None |
| TKT | A8K089 | Transketolase | Failure | 0 | None |
| SLIT2 | O94813 | Slit homolog 2 protein | Failure | 0 | None |
| LTB4R | Q15722 | Leukotriene B4 receptor 1 | Failure | 0 | None |
| FGG | P02679 | Fibrinogen gamma chain | Failure | 0 | None |
| TFRC | D3DXB0 | Transferrin receptor protein 1 | Failure | 0 | None |
| PSPH | P78330 | Phosphoserine phosphatase | Failure | 0 | None |
| GRIA2 | P42262 | Glutamate receptor 2 | Failure | 0 | None |
| EPHB1 | P54762 | Ephrin type-B receptor 1 | Failure | 0 | None |
| TAS2R5 | A4D1U0 | Taste receptor type 2 | Failure | 0 | None |
| TAAR9 | Q96RI9 | Trace amine-associated receptor 9 | Failure | 0 | None |
| PRHOXNB | A6NGE7 | Putative 2-oxo-4-hydroxy-4-carboxy-5-ureidoimidazoline decarboxylase | Failure | 0 | None |
| PIK3C2A | O00443 | Phosphatidylinositol 4-phosphate 3-kinase C2 domain-containing subunit alpha | Failure | 0 | None |
| GGA2 | D3DWF0 | ADP-ribosylation factor binding protein GGA2 | Failure | 0 | None |
| CBR3 | O75828 | Carbonyl reductase [NADPH] 3 | Failure | 0 | None |
| TRIM29 | Q14134 | Tripartite motif-containing protein 29 | Failure | 0 | None |
| TRGC2 | P03986 | T-cell receptor gamma-2 chain C region | Failure | 0 | None |
| TMPRSS13 | Q9BYE2 | Transmembrane protease serine 13 | Failure | 0 | None |
| PTPN23 | A8K0D7 | Tyrosine-protein phosphatase non-receptor type 23 | Failure | 0 | None |
| NME8 | Q8N427 | Thioredoxin domain-containing protein 3 | Failure | 0 | None |
| MAPK8 | P45983 | Mitogen-activated protein kinase 8 | Failure | 0 | None |
| HSPA4L | O95757 | Heat shock 70 kDa protein 4L | Failure | 0 | None |
| RNF128 | Q6PH80 | E3 ubiquitin-protein ligase RNF128 | Failure | 0 | None |
| HDAC6 | Q9UBN7 | Histone deacetylase 6 | Failure | 0 | None |
| CD4 | B2R737 | T-cell surface glycoprotein CD4 | Failure | 0 | None |
| BHMT2 | Q9H2M3 | S-methylmethionine--homocysteine S-methyltransferase BHMT2 | Failure | 0 | None |

**Continued columns**

| **OFFICIAL_GENE_SYMBOL** | **Structure** | **E-value** | **The binding sites** |
| --- | --- | --- | --- |
| EXOSC3 | NP_057126.2_17-275_100.000.pdb_A | 0.007 | 129 164 180 146 |
| MAPK12 | 1CM8.pdb_A | 0.008 | 273 276 217 214 213 226 |
| ITGAL | 1MQ8.pdb_B | 0.004 | 132 168 299 161 156 |
| PTGS1 | NP_000953.2_32-582_93.907.pdb_A | 4.458E-12 | 115 119 348 351 352 354 358 522 526 530 |
| PLA2G10 | 1LE6.pdb_A | 0.00004969 | 5 27 28 43 46 47 |
| FBP1 | NP_000498.2_10-337_99.687.pdb_A | 0.004 | 185 177 180 181 17 |
| CUL4B | 4A64.pdb_B | 0.006 | 443 483 482 504 |
| PTGS2 | NP_000954.1_18-569.pdb | 1.988E-13 | 102 106 335 338 339 341 345 513 517 |
| MMP12 | 3BA0.pdb_A | 0.006 | 462 468 470 469 282 |
| CDK13 | NP_112557.2_704-1013_46.715.pdb_A | 0.0002773 | 838 824 828 829 856 |
| TNFAIP6 | 2PF5.pdb_B | 0.0007668 | 68 69 47 78 |
| PLA2G3 | NP_056530.2_151-283_34.815.pdb_A | 0.003 | 159 160 181 184 185 217 |
| HLA-A | 3HG1.pdb_A | 0.005 | 266 213 205 203 259 |
| MOCS3 | 3I2V.pdb_A | 0.005 | 16 15 14 35 |
| AIDA | NP_073742.2_152-305_99.351.pdb_A | 0.0003293 | 220 181 184 182 243 |
| RAC1 | 1RYH.pdb_A | 0.003 | 56 124 123 97 |
| PLA2G5 | NP_000920.1_21-138_49.167.pdb_A | 9.617E-16 | 22 29 47 48 49 64 67 68 |
| PLA2G1B | NP_000919.1_25-146_56.911.pdb_A | 4.495E-19 | 27 31 50 51 52 67 70 71 91 128 |
| PLA2G2A | NP_000291.1_21-144_100.000.pdb_A | 6.978E-14 | 22 25 29 48 49 64 67 68 118 |
| TNFSF14 | 4EN0.pdb_A | 0.008 | 224 162 154 153 187 |
| CHIT1 | 1WB0.pdb_A | 6.654E-20 | 29 31 35 295 360 361 363 364 |
| EGFLAM | NP_877950.1_586-775_35.263.pdb_A | 0.005 | 774 772 773 745 |
| PLA2G2D | NP_036532.1_21-145_51.587.pdb_A | 0.000007796 | 25 48 49 64 67 68 82 |
| MICAL2 | NP_055447.1_16-488_59.660.pdb_A | 0.007 | 448 411 410 97 398 |
| PLA2G2F | NP_073730.3_64-195_40.164.pdb_A | 4.833E-08 | 65 84 90 91 92 107 110 111 |
| LPO | NP_006142.1_118-712_86.013.pdb | 2.156E-19 | 222 226 372 375 498 538 539 540 541 |
| SERPING1 | NP_000053.2_125-498_99.458.pdb_A | 0.0003732 | 209 205 130 244 |
| RNF125 | NP_060301.2_9-123_29.412.pdb_A | 0.0005886 | 56 61 52 53 75 |
| TRIM7 | NP_203128.1_7-135_29.915.pdb_A | 0.0006474 | 48 53 44 45 81 |
| NCEH1 | NP_065843.3_60-440_28.155.pdb_A | 0.0007343 | 68 412 411 377 380 |
| LDLR | 2FCW.pdb_B | 0.0007806 | 113 116 95 96 97 93 |
| SKP1 | NP_733779.1_2-154_100.000.pdb_A | 0.001 | 107 113 62 65 |
| NFATC2 | 2AS5.pdb_M | 0.001 | 591 593 588 673 |
| GAA | NP_000143.2_89-952_45.930.pdb_A | 0.001 | 647 648 658 687 |
| VRL2 | NP_001036189.1_34-346_55.814.pdb_A | 0.002 | 155 140 152 54 55 |
| PVRL2 | NP_002847.1_34-346_55.814.pdb_A | 0.002 | 155 140 152 54 55 |
| PIK3CA | 3HHM.pdb_A | 0.002 | 954 907 903 984 1039 |
| NQO2 | NP_000895.2_3-230_99.571.pdb_A | 0.002 | 127 180 181 183 174 |
| CPA2 | 1AYE.pdb_A | 0.002 | 250 244 207 210 |
| CLK2 | NP_003984.2_142-489_71.676.pdb_A | 0.002 | 366 472 470 281 282 |
| CCNA2 | NP_001228.1_175-432_100.000.pdb_A | 0.002 | 360 359 337 413 319 |
| ATPBD4 | NP_542381.1_3-251_41.964.pdb_A | 0.002 | 219 15 14 10 215 |
| ARHGAP5 | NP_001164.2_1244-1455_100.000.pdb_A | 0.002 | 1336 1335 1331 1280 1276 |
| ADAM17 | 2DDF.pdb_A | 0.002 | 421 423 432 453 416 |
| ABCB11 | NP_003733.2_45-1315_53.254.pdb_A | 0.002 | 987 986 981 984 982 |
| LEP | 1AX8.pdb_A | 0.003 | 3 146 145 96 |
| HS3ST2 | NP_006034.1_111-367_43.725.pdb_A | 0.003 | 305 313 315 325 |
| CTSF | 1M6D.pdb_A | 0.003 | 173 171 200 201 159 158 |
| CDKL2 | 4AAA.pdb_A | 0.003 | 127 113 117 118 145 |
| TLE3 | NP_001098662.1_331-769_94.985.pdb_A | 0.004 | 553 600 589 598 591 |
| PLXNA2 | NP_079455.3_62-508_27.293.pdb_A | 0.004 | 184 129 130 137 157 |
| IGDCC4 | NP_066013.1_42-422_31.215.pdb_A | 0.004 | 322 323 310 312 |
| HEPH | NP_055614.1_112-797_38.863.pdb_A | 0.004 | 295 293 267 264 |
| FCAR | NP_579808.1_12-193_34.756.pdb_A | 0.004 | 66 18 37 88 |
| TKT | 3MOS.pdb_A | 0.005 | 224 228 52 49 183 71 |
| SLIT2 | NP_004778.1_28-259_41.489.pdb_A | 0.005 | 224 218 212 213 236 |
| LTB4R | Q15722.pdb_A | 0.005 | 88 90 89 168 94 |
| FGG | NP_000500.2_31-421_100.000.pdb_A | 0.005 | 365 392 352 356 348 |
| TFRC | 1SUV.pdb_A | 0.006 | 576 558 490 556 |
| PSPH | NP_004568.2_4-225_99.554.pdb_A | 0.006 | 211 193 192 173 18 |
| GRIA2 | 2WJW.pdb_A | 0.006 | 100 77 76 78 330 103 |
| EPHB1 | NP_004432.1_16-527_41.822.pdb_A | 0.006 | 281 259 255 267 203 |
| TAS2R5 | A4D1U0.pdb_A | 0.007 | 276 226 225 268 |
| TAAR9 | NP_778227.3_36-335_38.889.pdb_A | 0.007 | 123 168 117 118 116 |
| PRHOXNB | NP_001099047.1_1-168_52.071.pdb_A | 0.007 | 28 62 66 67 |
| PIK3C2A | NP_002636.2_1042-1391_42.135.pdb_A | 0.007 | 1371 1375 1237 1239 1238 1289 |
| GGA2 | 1MHQ.pdb_B | 0.007 | 29 49 93 92 |
| CBR3 | NP_001227.1_4-277_71.942.pdb_A | 0.007 | 147 146 143 195 158 |
| TRIM29 | NP_036233.2_168-262_40.404.pdb_A | 0.008 | 245 244 247 252 |
| TRGC2 | 1HXM.pdb_D | 0.008 | 100 98 97 23 |
| TMPRSS13 | NP_001070731.1_236-561_39.879.pdb_A | 0.008 | 400 351 352 367 369 |
| PTPN23 | 3RAU.pdb_B | 0.008 | 11 130 133 188 |
| NME8 | NP_057700.3_10-110_25.743.pdb_A | 0.008 | 44 42 63 33 |
| MAPK8 | NP_002741.1_7-364_100.000.pdb_A | 0.008 | 74 352 61 67 101 |
| HSPA4L | NP_055093.2_3-568_30.035.pdb_A | 0.008 | 26 373 376 375 380 |
| RNF128 | 3ICU.pdb_A | 0.009 | 145 128 129 126 96 |
| HDAC6 | NP_006035.2_481-835_49.438.pdb_A | 0.009 | 576 577 570 572 622 |
| CD4 | 1WIO.pdb_B | 0.009 | 24 18 16 84 27 |
| BHMT2 | NP_060084.2_10-362_77.684.pdb_A | 0.009 | 262 293 290 208 258 |

**Note: Docking result**: This column shows the results of molecular docking, Success or Failure; **Number of Docking pose**: If molecular docking is success, the number of docking pose was recorded; **ΔG^PB^_binding_** :This column shows the calculated binding free energies; **Structure** :This column shows the protein structures used in this study; **E-value** : This column shows the E-value which menas the probability of getting the binding site by chance.
