# Supplementary material for: Proteome-wide prediction of targets for aspirin: new insight into the molecular mechanism of aspirin
Source: PeerJ. 2016 Mar 10;4:e1791. doi: 10.7717/peerj.1791 (PMC4793309; doi:10.7717/peerj.1791)
Supplement: Table S2 [file peerj-04-1791-s002.docx]

**Table S2 –The coordinates of 23 putative binding pockets**

| **Gene name** | **UniProtKB entry** | **Structure** | **The coordinates of BSiteAs center** |
| --- | --- | --- | --- |
| EXOSC3 | Q9NQT5 | NP_057126_EXOSC3.pdb | 97.269,37.103,160.231 |
| MAPK12 | P53778 | 1CM8_MAPK12.pdb | 28.210,75.363,23.194 |
| ITGAL | O43746 | 1MQ8_ITGAL.pdb | 21.839,18.336,32.590 |
| PTGS2 | [P35354](http://www.uniprot.org/uniprot/P35354) | NP_000954_PTGS2.pdb | 56.496,45.908,81.461 |
| PTGS1 | P23219 | NP_000953_PTGS1.pdb | 67.267,19.737,188.894 |
| PLA2G10 | O15496 | 1LE6_PLA2G10.pdb | 19.007,2.842,27.747 |
| FBP1 | P09467 | NP_000498_FBP1.pdb | -1.842,85.780,14.257 |
| CUL4B | Q13620 | 4A64_CUL4B.pdb | 18.895,-12.467,83.165 |
| MMP12 | P39900 | 3BA0_MMP12.pdb | -21.079,-34.484,6.282 |
| CDK13 | Q14004 | NP_112557_CDK13.pdb | 15.299,3.964,19.965 |
| TNFAIP6 | P98066 | 2PF5_TNFAIP6.pdb | -31.542,17.441,17.512 |
| PLA2G3 | Q9NZ20 | NP_056530_PLA2G3.pdb | 41.973,32.399,19.233 |
| HLA-A | O19619 | 3HG1_HLA-A.pdb | 74.439,-33.845,24.089 |
| MOCS3 | O95396 | 3I2V_MOCS3.pdb | 13.640,2.188,1.656 |
| AIDA | Q96BJ3 | NP_073742_AIDA.pdb | -5.971,7.171,35.190 |
| RAC1 | P63000 | 1RYH_RAC1.pdb | -14.280,56.898,33.909 |
| PLA2G5 | P39877 | NP_000920_PLA2G5.pdb | 25.566,46.132,36.014 |
| PLA2G1B | P04054 | NP_000919_PLA2G1B.pdb | 34.828,75.076,52.890 |
| PLA2G2A | P14555 | NP_000291_PLA2G2A.pdb | 58.519,7.465,73.921 |
| TNFSF14 | O43557 | 4EN0_TNFSF14.pdb | -8.260,12.988,4.768 |
| CHIT1 | Q13231 | 1WB0.pdb_A | 17.7787,-6.21062,15.51161 |
| EGFLAM | Q63HQ2 | NP_877950_EGFLAM.pdb | -16.190,-4.145,-0.769 |
| PLA2G2D | Q9UNK4 | NP_036532_PLA2G2D.pdb | -43.355,137.500,54.861 |
