# Supplementary material for: Proteome-wide prediction of targets for aspirin: new insight into the molecular mechanism of aspirin
Source: PeerJ. 2016 Mar 10;4:e1791. doi: 10.7717/peerj.1791 (PMC4793309; doi:10.7717/peerj.1791)
Supplement: Table S3 [file peerj-04-1791-s003.docx]

**Table S3. The result of prediction of aspirin targets by PharmMapper tool.**

| **Pharma Model** | **Fit** | **Name** | **Class** |
| --- | --- | --- | --- |
| 1r1h_v | 4.278 | Neprilysin | HYDROLASE |
| 5p21_v | 3.999 | GTPase HRas | ONCOGENE PROTEIN |
| 2aeb_v | 3.995 | Arginase-1 | HYDROLASE |
| 4gss_v | 3.859 | Glutathione S-transferase P | TRANSFERASE |
| 1i44_v | 3.811 | Insulin receptor | TRANSFERASE |
| 1kav_v | 3.81 | Tyrosine-protein phosphatase non-receptor type 1 | HYDROLASE |
| 1hfv_v | 3.775 | NONE | NONE |
| 1p2s_v | 3.758 | GTPase HRas | SIGNALING PROTEIN |
| 1q1z_v | 3.746 | Sulfotransferase family cytosolic 2B member 1 | NONE |
| 1m51_v | 3.739 | Phosphoenolpyruvate carboxykinase, cytosolic [GTP] | NONE |
| 1o4h_v | 3.732 | Proto-oncogene tyrosine-protein kinase Src | SIGNALING PROTEIN |
| 1hy7_v | 3.693 | Stromelysin-1 | HYDROLASE |
| 1uzf_v | 3.687 | Angiotensin-converting enzyme | METALLOPROTEASE |
| 18gs_v | 3.686 | Glutathione S-transferase P | TRANSFERASE |
| 1pgt_v | 3.682 | Glutathione S-transferase P | TRANSFERASE |
| 1jf7_v | 3.625 | Tyrosine-protein phosphatase non-receptor type 1 | HYDROLASE |
| **1db4_v** | **3.617** | **Phospholipase A2, membrane associated** | **NONE** |
| 1hti_v | 3.617 | Triosephosphate isomerase | ISOMERASE(INTRAMOLECULAR |
| 1awb_v | 3.615 | Inositol monophosphatase | HYDROLASE |
