# Supplementary material for: Proteome-wide prediction of targets for aspirin: new insight into the molecular mechanism of aspirin
Source: PeerJ. 2016 Mar 10;4:e1791. doi: 10.7717/peerj.1791 (PMC4793309; doi:10.7717/peerj.1791)
Supplement: Figure S1 [file peerj-04-1791-s005.pdf]

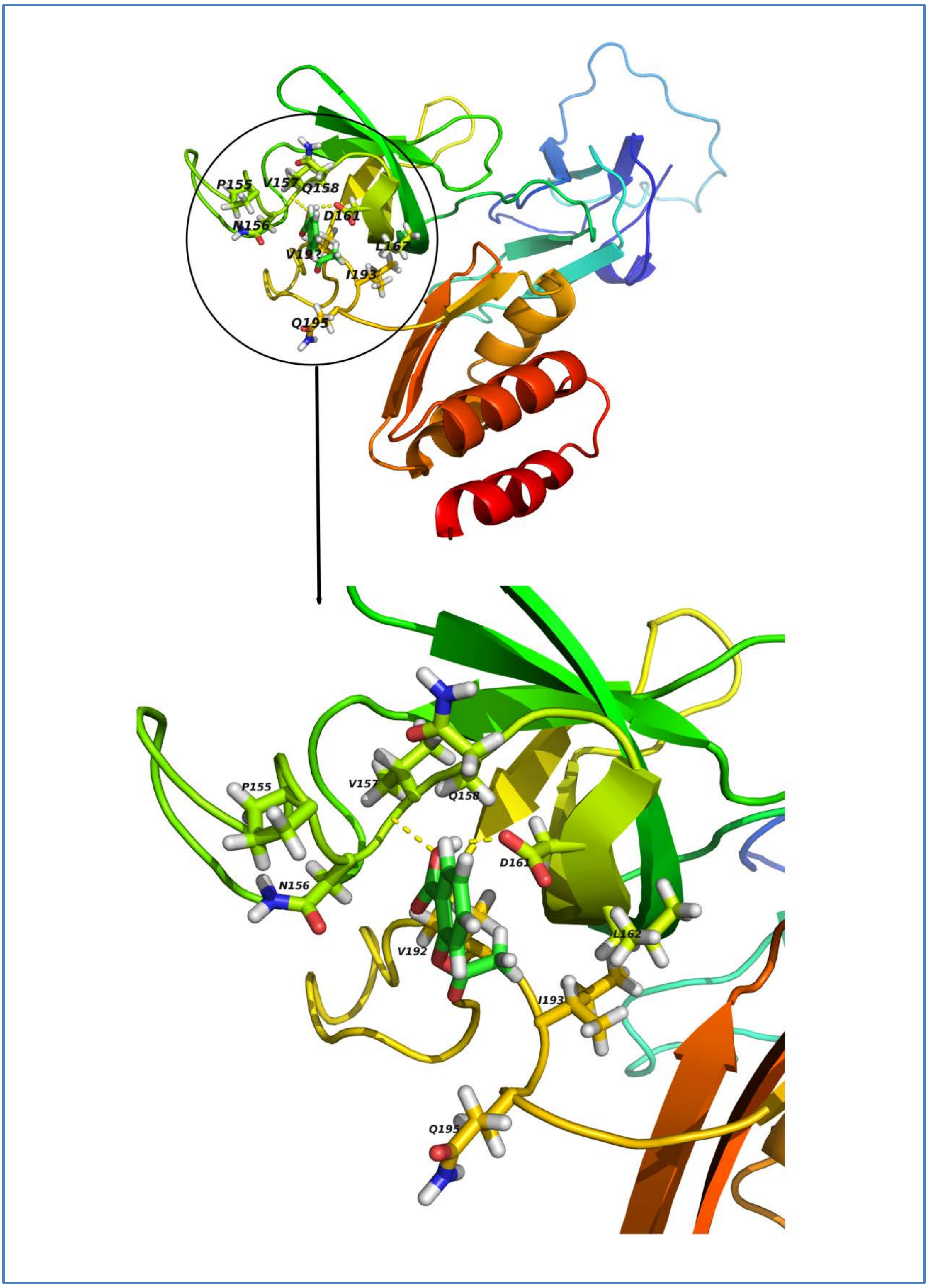

Figure S1. The binding mode of aspirin to the protein Exosome complex component RRP40 (EXOSC3). The overview and close-up view of the binding mode of aspirin to EXOSC3 were shown in the top and bottom panels, respectively. Secondary structural elements were colored from blue (N-terminus) to red (C-terminus). The compound aspirin is colored with green. The residues lying within 5 Ǻ from atoms of the aspirin are shown as sticks. The H-bonds are shown by dashed yellow line.

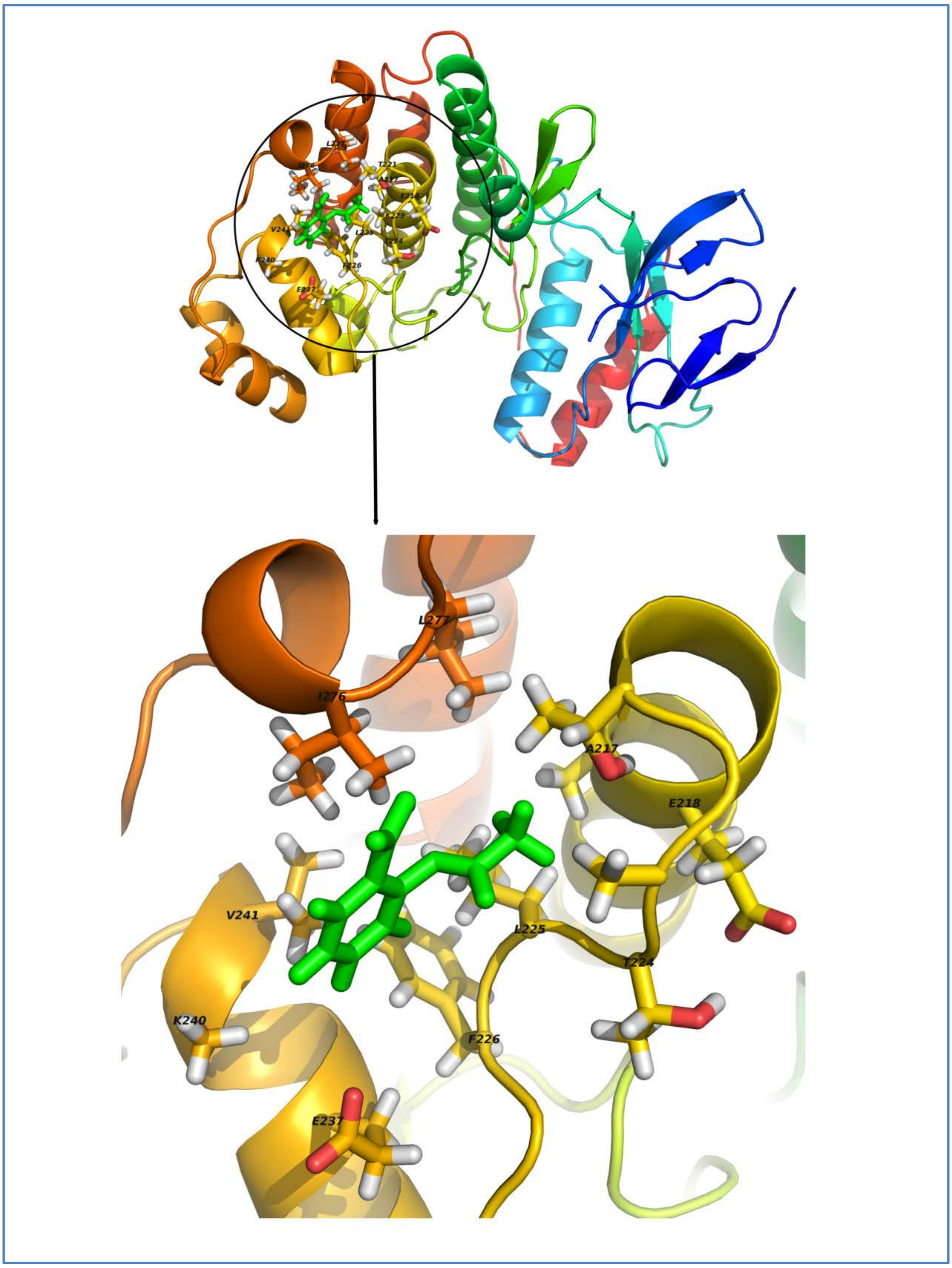

Figure S2. The binding mode of aspirin to the protein Mitogen-activated protein kinase 12 (MAPK12). The overview and close-up view of the binding mode of aspirin to MAPK12 were shown in the top and bottom panels, respectively. Secondary structural elements were colored from blue (N-terminus) to red (C-terminus). The compound aspirin is colored with green. The residues lying within 5 Ǻ from atoms of the aspirin are shown as sticks. The H-bonds are shown by dashed yellow line.

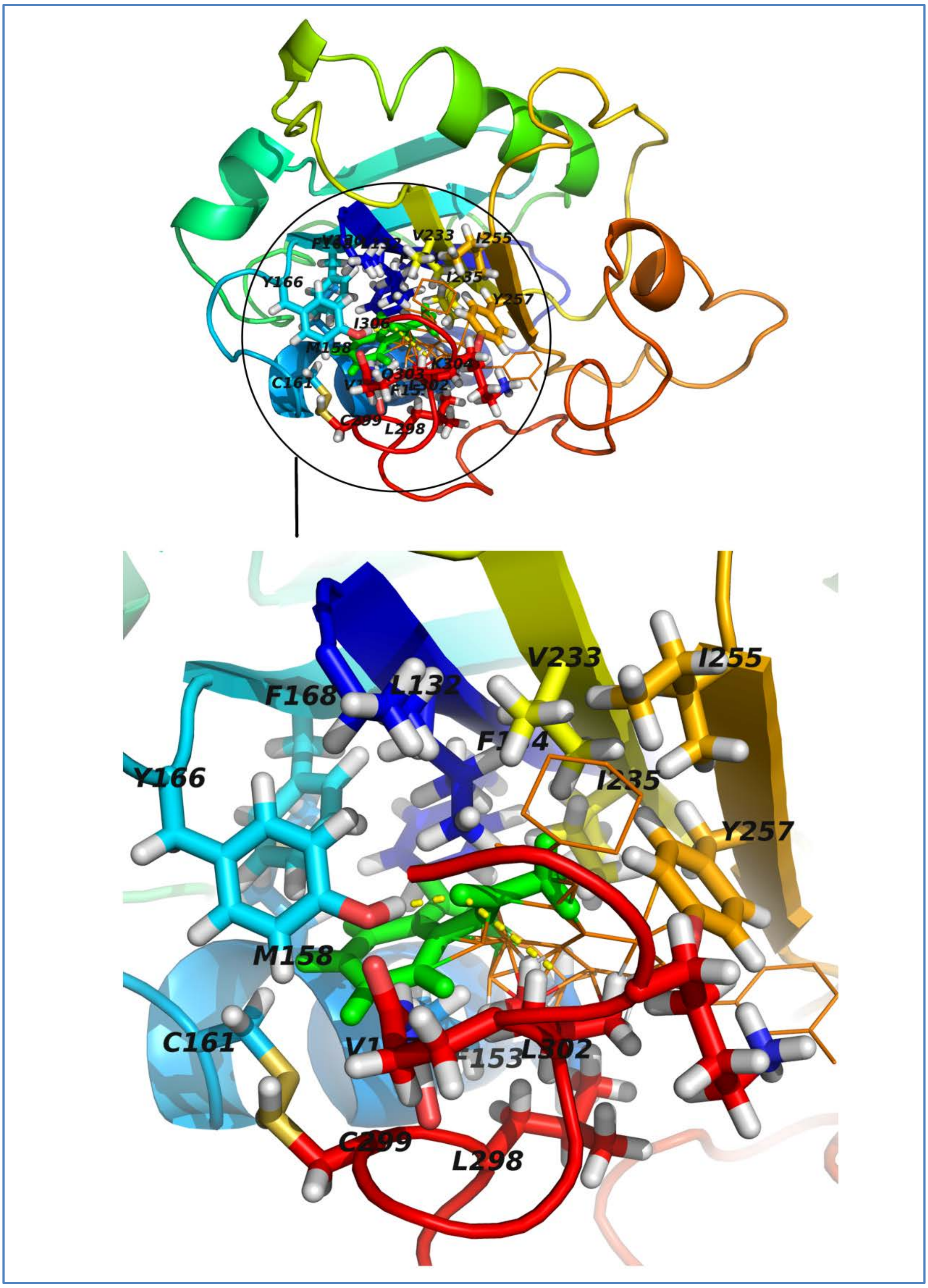

Figure S3. The binding mode of aspirin to the protein Integrin alpha-L (ITGAL). The overview and close-up view of the binding mode of aspirin to ITGAL were shown in the top and bottom panels, respectively. Secondary structural elements were colored from blue (N-terminus) to red (C-terminus). The compound aspirin is colored with green. The residues lying within 5 Ǻ from atoms of the aspirin are shown as sticks. The H-bonds are shown by dashed yellow line.

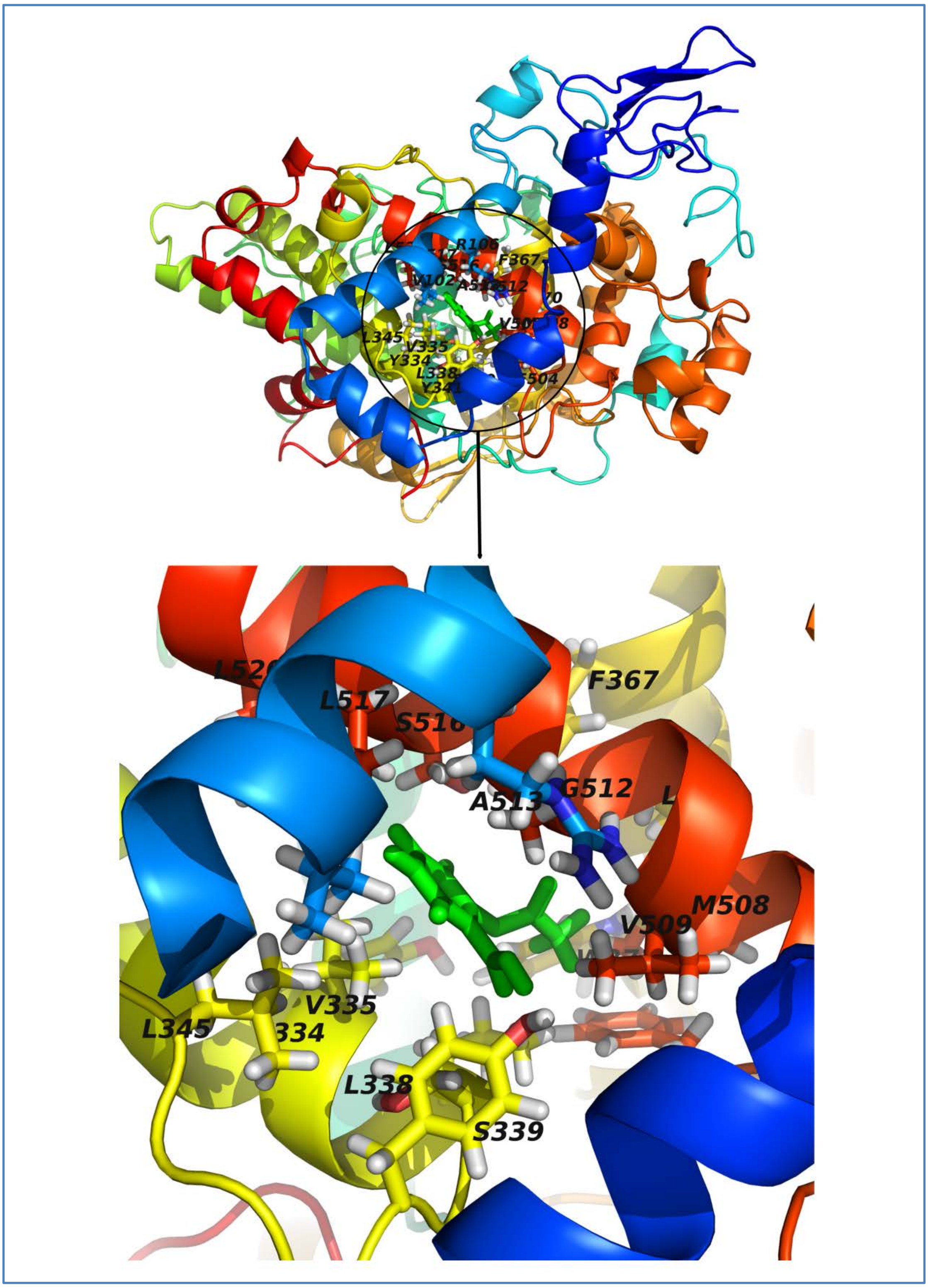

Figure S4. The binding mode of aspirin to the protein Prostaglandin G/H synthase 2 (PTGS2). The overview and close-up view of the binding mode of aspirin to PTGS2 were shown in the top and bottom panels, respectively. Secondary structural elements were colored from blue (N-terminus) to red (C-terminus). The compound aspirin is colored with green. The residues lying within 5 Ǻ from atoms of the aspirin are shown as sticks. The H-bonds are shown by dashed yellow line.

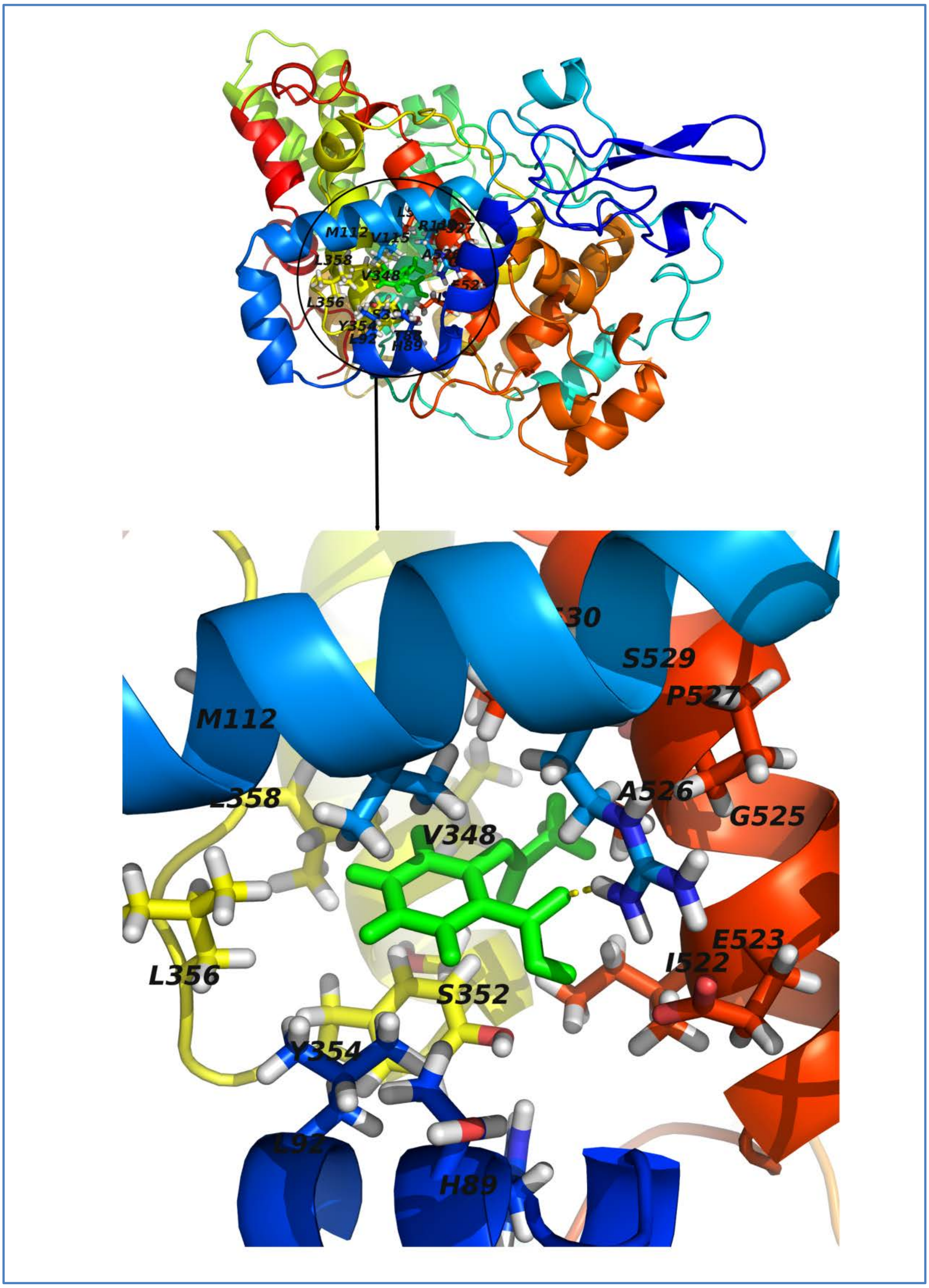

Figure S5. The binding mode of aspirin to the protein Prostaglandin G/H synthase 1 (PTGS1). The overview and close-up view of the binding mode of aspirin to PTGS1 were shown in the top and bottom panels, respectively. Secondary structural elements were colored from blue (N-terminus) to red (C-terminus). The compound aspirin is colored with green. The residues lying within 5 Ǻ from atoms of the aspirin are shown as sticks. The H-bonds are shown by dashed yellow line.

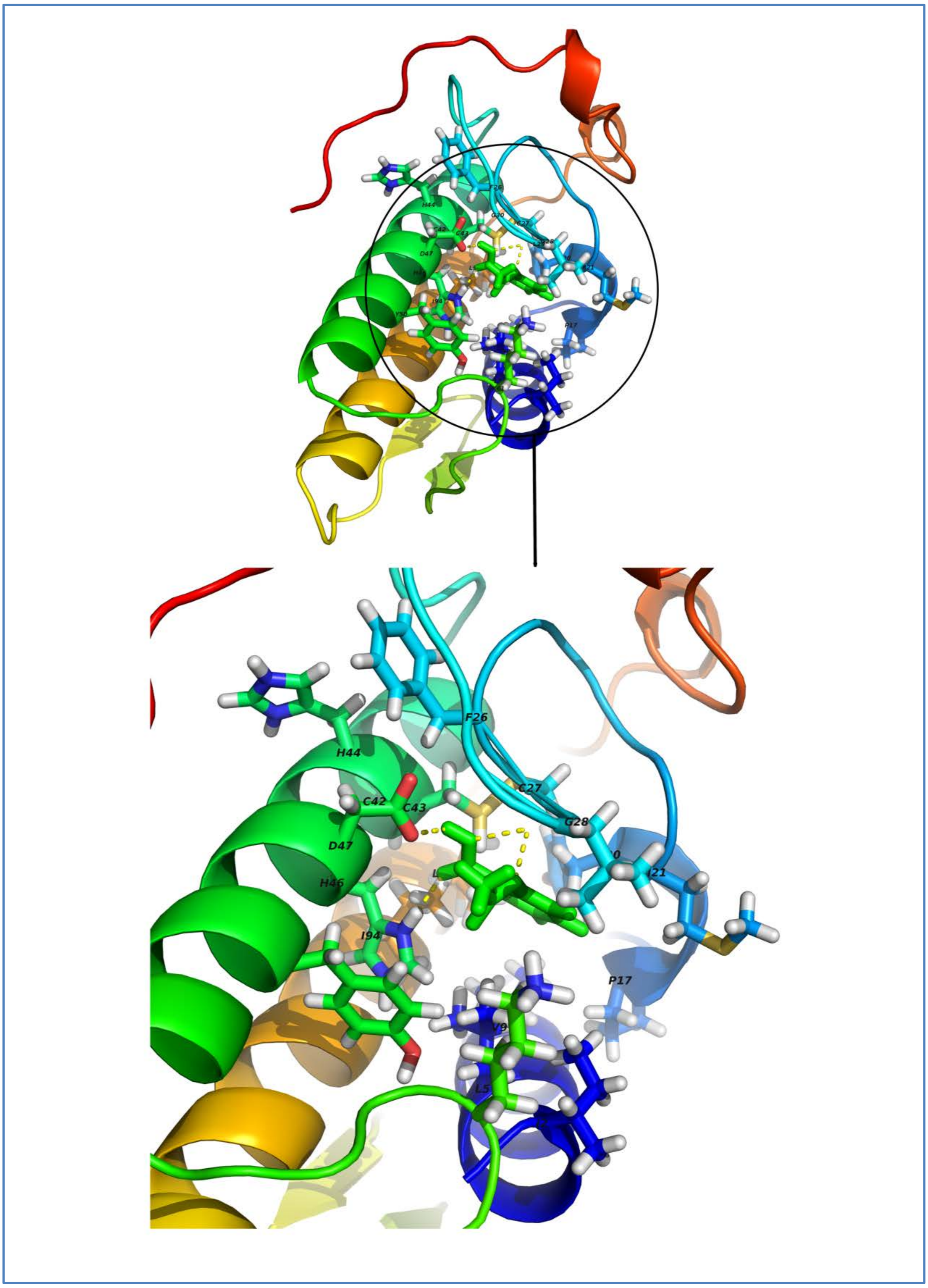

Figure S6. The binding mode of aspirin to the protein Group X Secretory Phospholipase A2 (PLA2G10). The overview and close-up view of the binding mode of aspirin to PLA2G10 were shown in the top and bottom panels, respectively. Secondary structural elements were colored from blue (N-terminus) to red (C-terminus). The compound aspirin is colored with green. The residues lying within 5 Ǻ from atoms of the aspirin are shown as sticks. The H-bonds are shown by dashed yellow line.

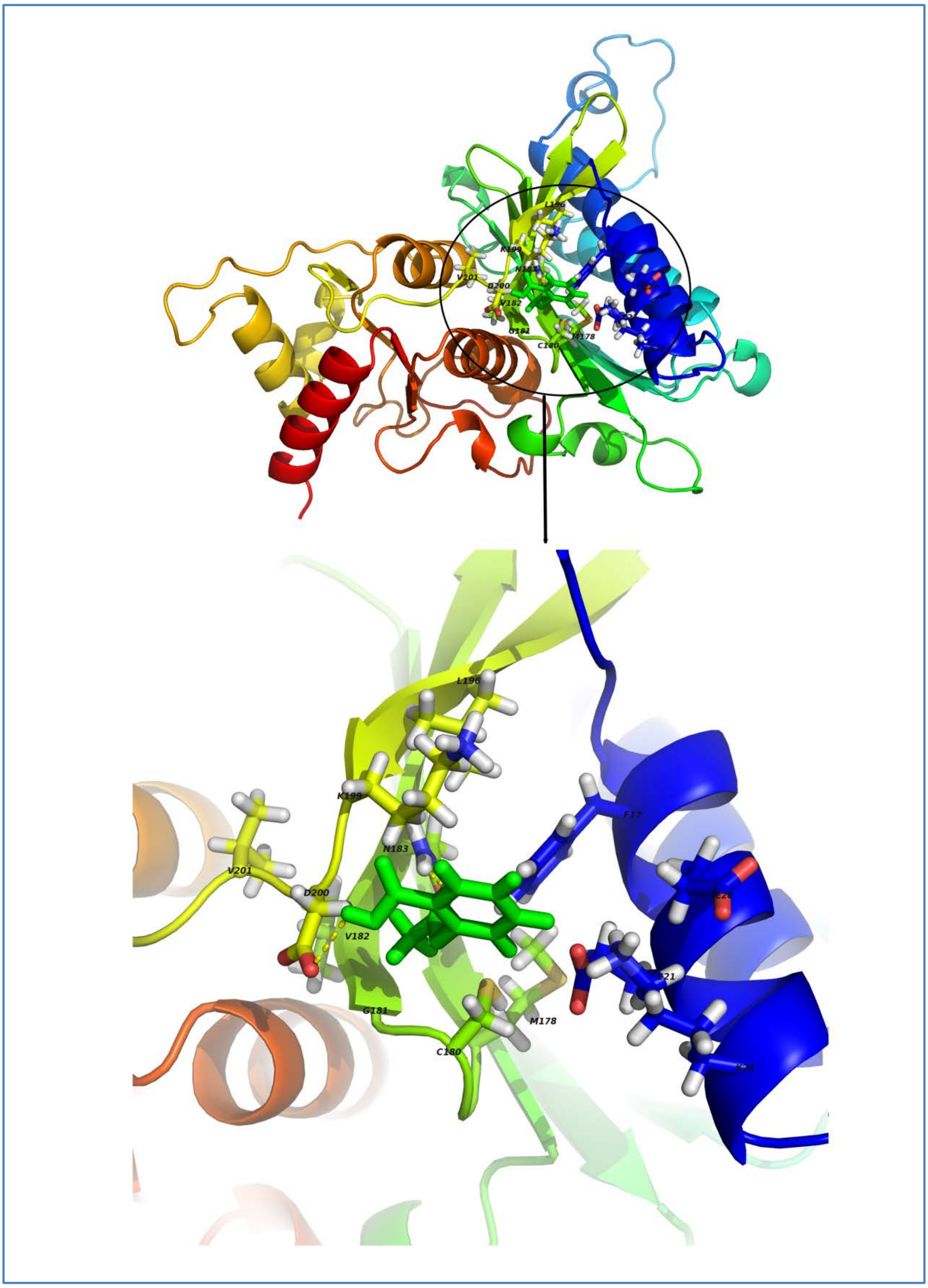

Figure S7. The binding mode of aspirin to the protein Fructose-1,6-bisphosphatase 1 (FBP1). The overview and close-up view of the binding mode of aspirin to FBP1 were shown in the top and bottom panels, respectively. Secondary structural elements were colored from blue (N-terminus) to red (C-terminus). The compound aspirin is colored with green. The residues lying within 5 Ǻ from atoms of the aspirin are shown as sticks. The H-bonds are shown by dashed yellow line.

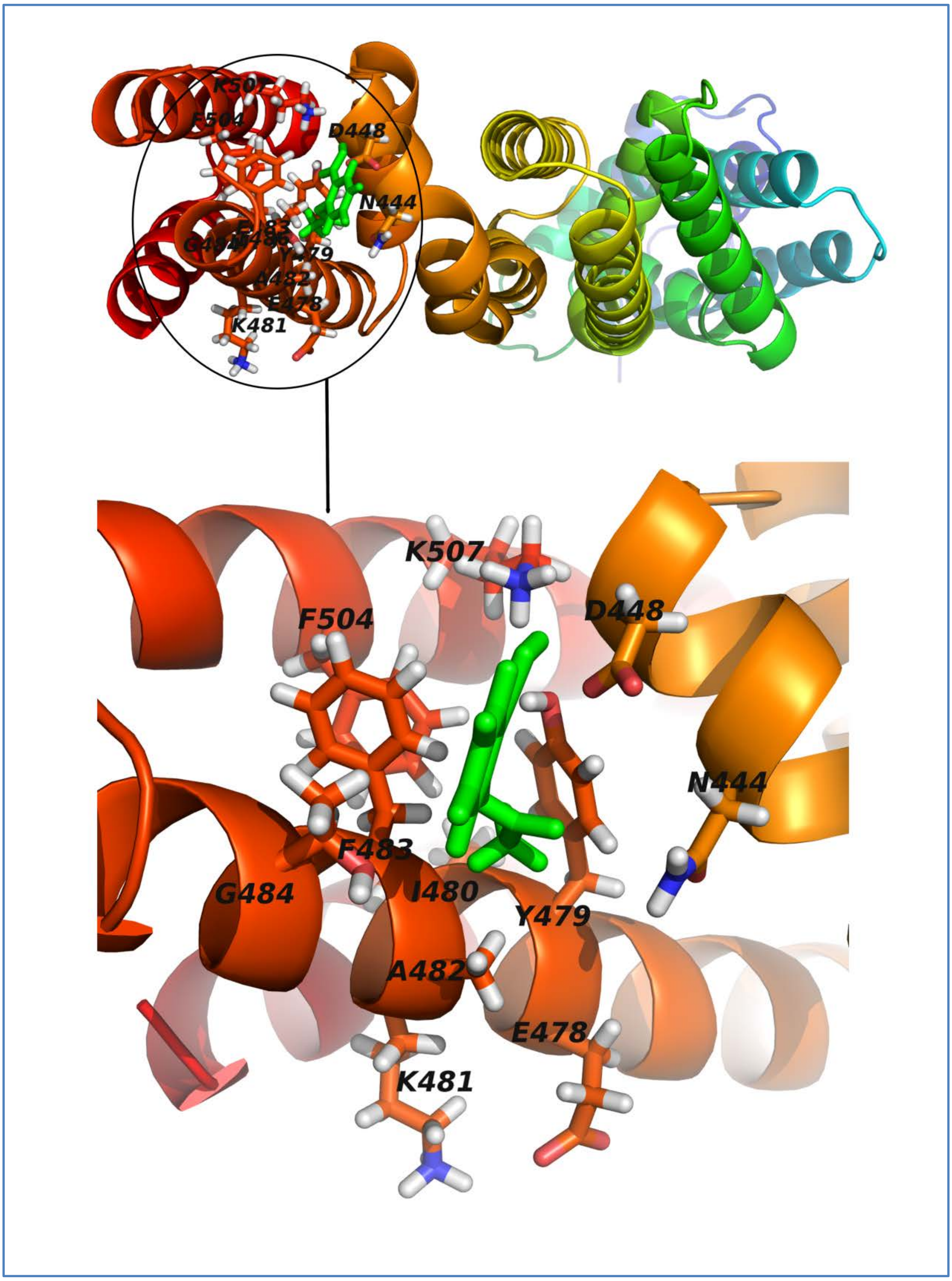

Figure S8. The binding mode of aspirin to the protein Cullin-4B (CUL4B). The overview and close-up view of the binding mode of aspirin to CUL4B were shown in the top and bottom panels, respectively. Secondary structural elements were colored from blue (N-terminus) to red (C-terminus). The compound aspirin is colored with green. The residues lying within 5 Ǻ from atoms of the aspirin are shown as sticks. The H-bonds are shown by dashed yellow line.

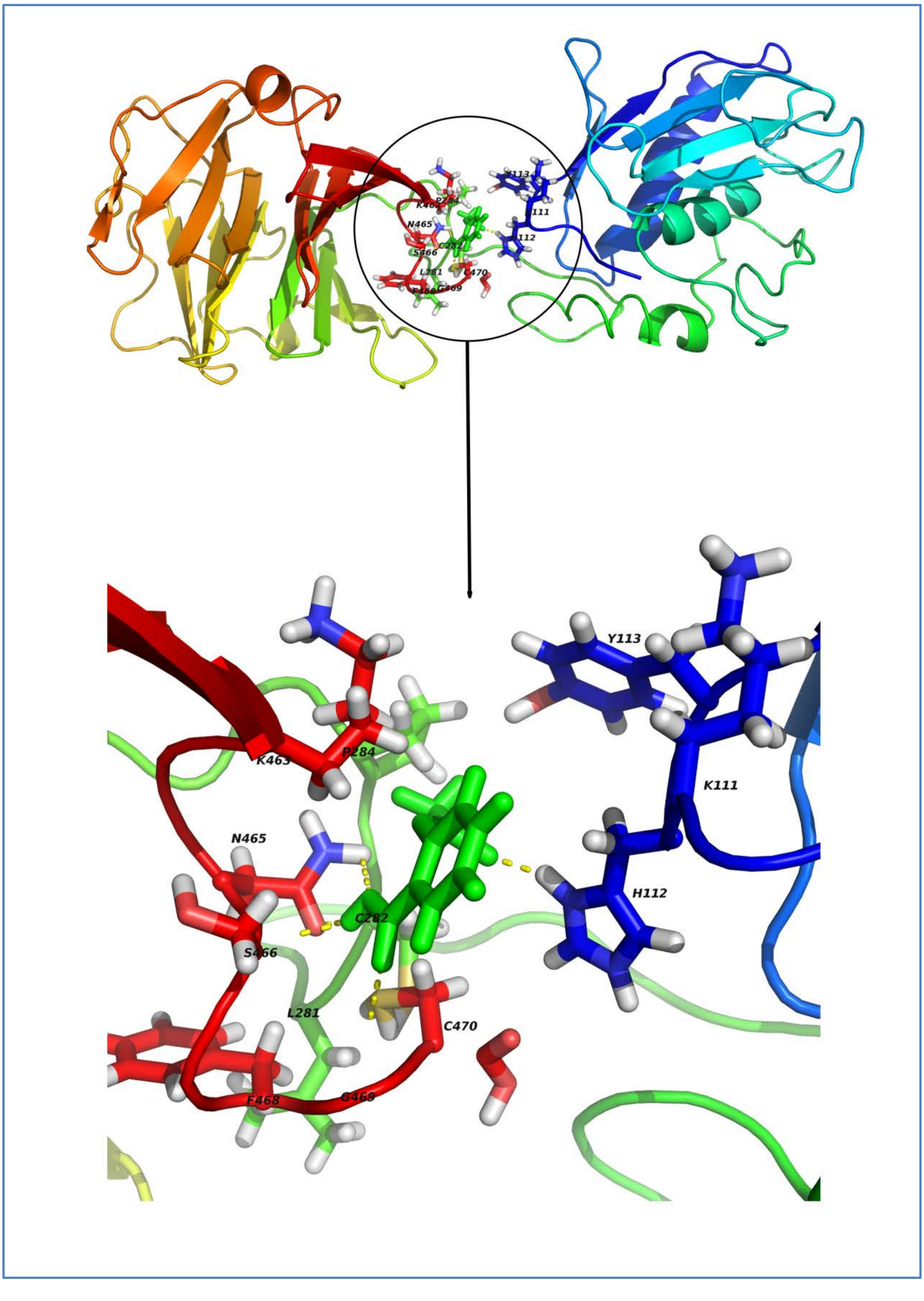

Figure S9. The binding mode of aspirin to the protein Macrophage metalloelastase (MMP12). The overview and close-up view of the binding mode of aspirin to MMP12 were shown in the top and bottom panels, respectively. Secondary structural elements were colored from blue (N-terminus) to red (C-terminus). The compound aspirin is colored with green. The residues lying within 5 Ǻ from atoms of the aspirin are shown as sticks. The H-bonds are shown by dashed yellow line.

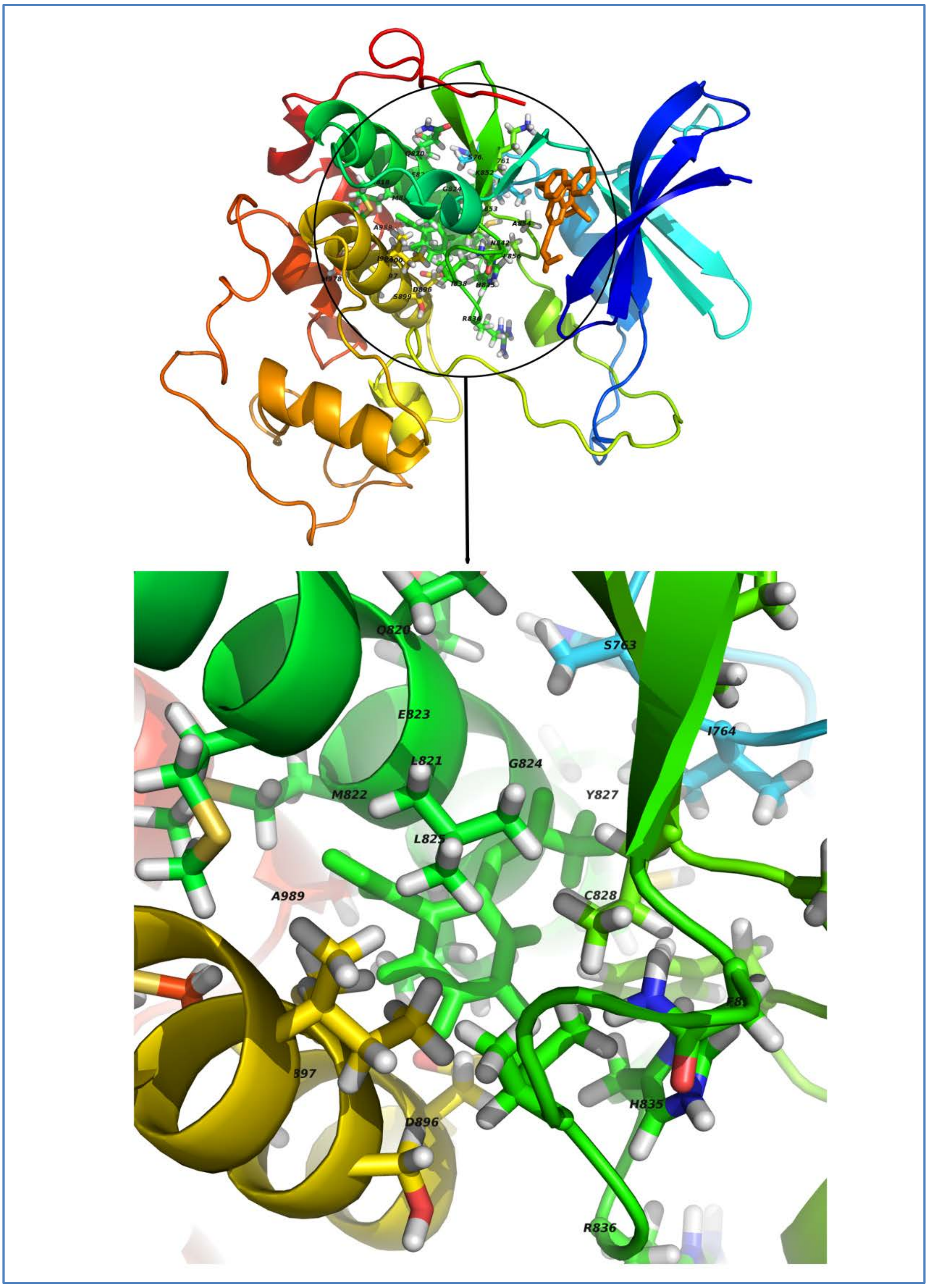

Figure S10. The binding mode of aspirin to the protein Cyclin-dependent kinase 13 (CDK13). The overview and close-up view of the binding mode of aspirin to CDK13 were shown in the top and bottom panels, respectively. Secondary structural elements were colored from blue (N-terminus) to red (C-terminus). The compound aspirin is colored with green. The residues lying within 5 Ǻ from atoms of the aspirin are shown as sticks. The H-bonds are shown by dashed yellow line.

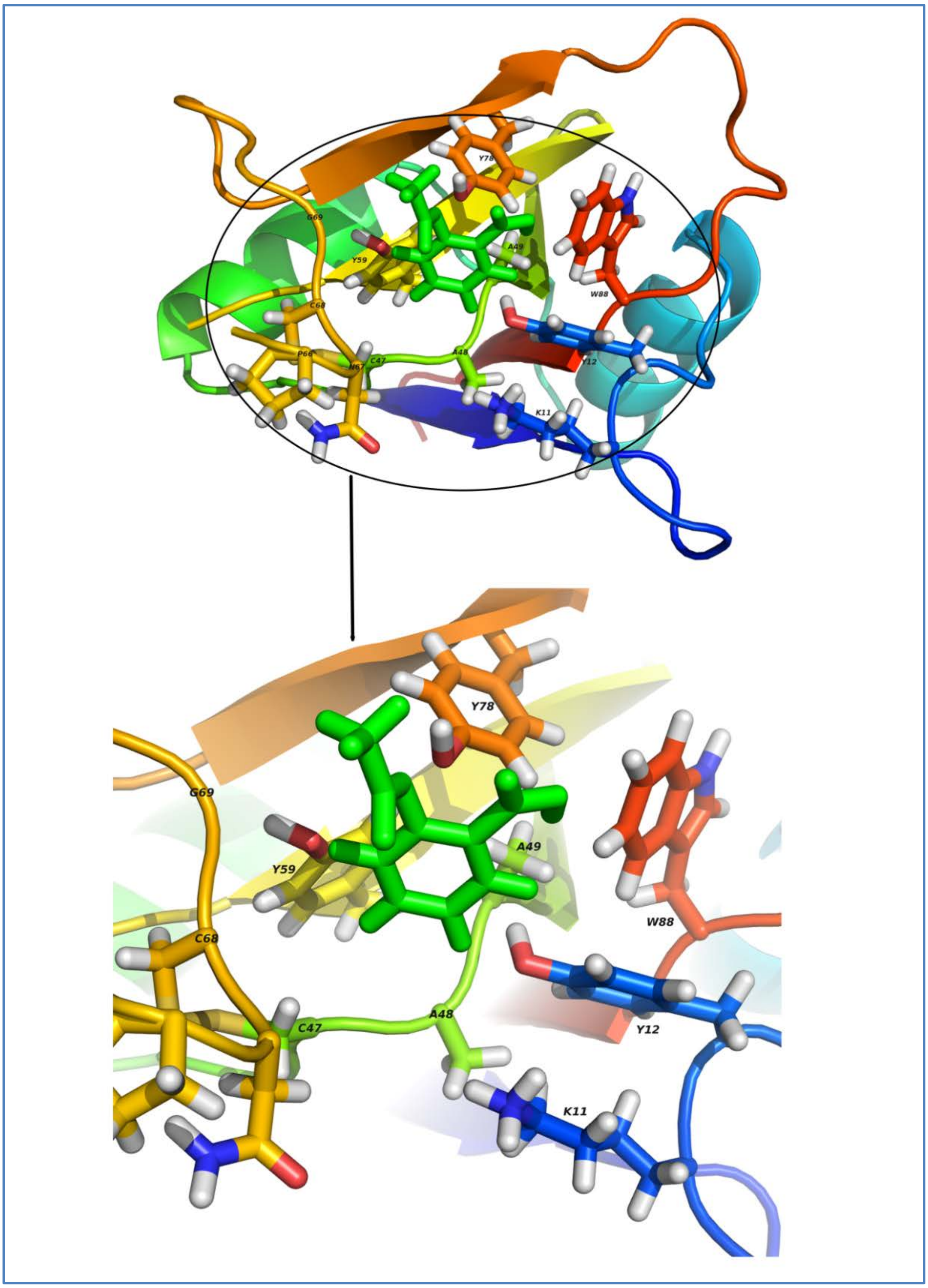

Figure S11. The binding mode of aspirin to the protein Tumor necrosis factor-inducible protein TSG-6 (TNFAIP6). The overview and close-up view of the binding mode of aspirin to TNFAIP6 were shown in the top and bottom panels, respectively. Secondary structural elements were colored from blue (N-terminus) to red (C-terminus). The compound aspirin is colored with green. The residues lying within 5 Ǻ from atoms of the aspirin are shown as sticks. The H-bonds are shown by dashed yellow line.

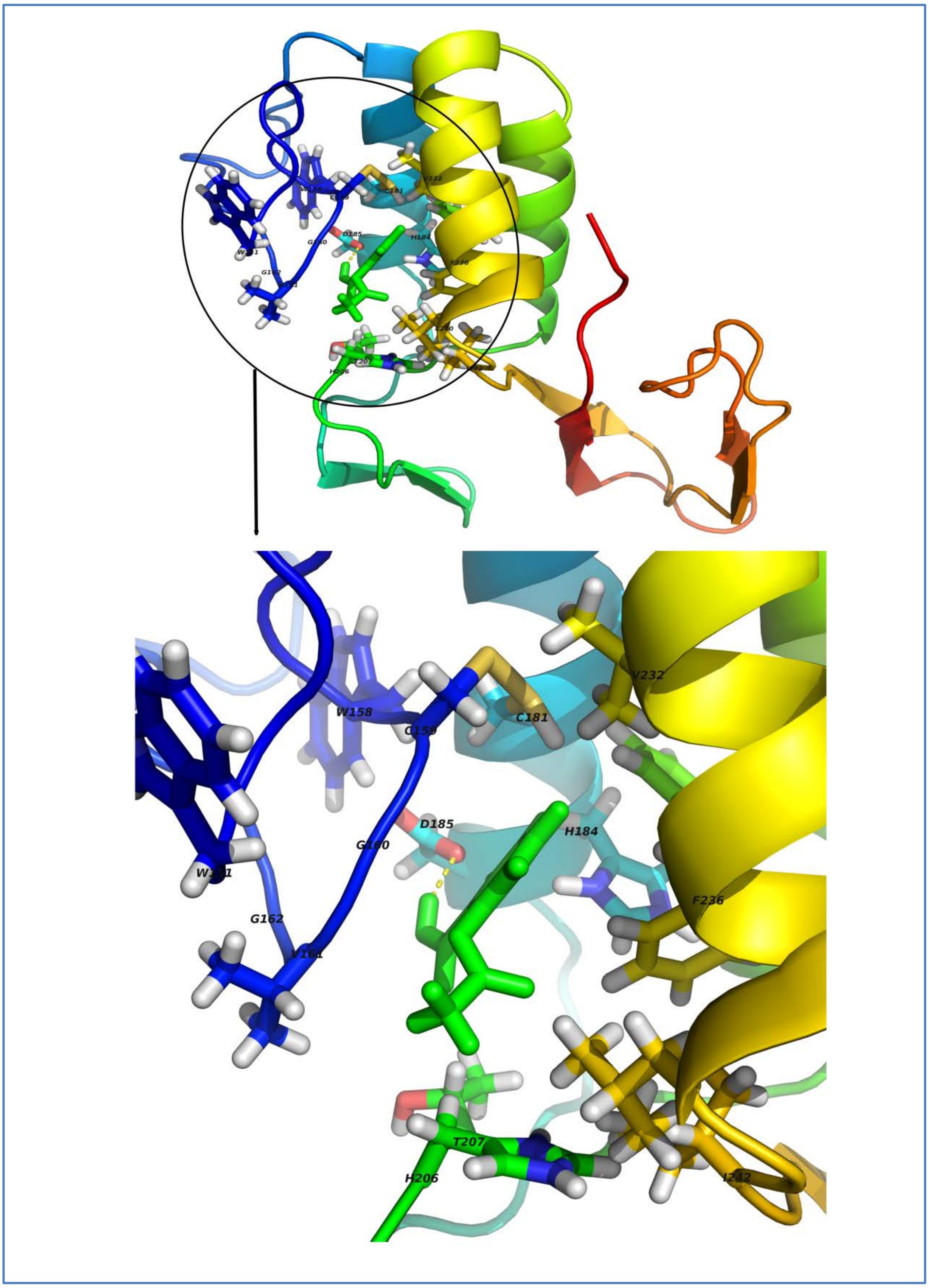

Figure S12. The binding mode of aspirin to the protein Group 3 secretory phospholipase A2 (PLA2G3). The overview and close-up view of the binding mode of aspirin to PLA2G3 were shown in the top and bottom panels, respectively. Secondary structural elements were colored from blue (N-terminus) to red (C-terminus). The compound aspirin is colored with green. The residues lying within 5 Ǻ from atoms of the aspirin are shown as sticks. The H-bonds are shown by dashed yellow line.

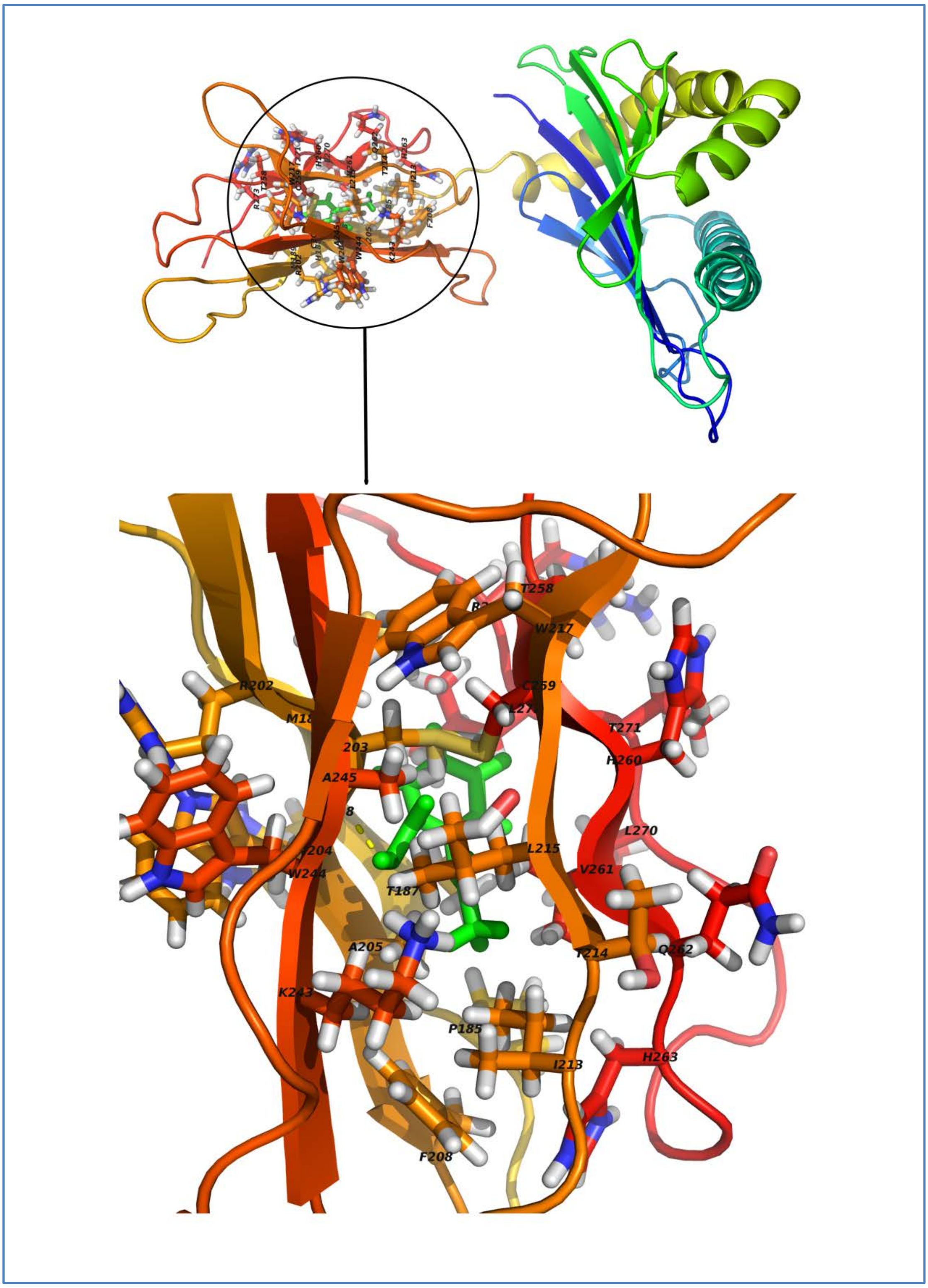

Figure S13. The binding mode of aspirin to the protein MHC class I antigen (HLA-A). The overview and close-up view of the binding mode of aspirin to HLA-A were shown in the top and bottom panels, respectively. Secondary structural elements were colored from blue (N-terminus) to red (C-terminus). The compound aspirin is colored with green. The residues lying within 5 Ǻ from atoms of the aspirin are shown as sticks. The H-bonds are shown by dashed yellow line.

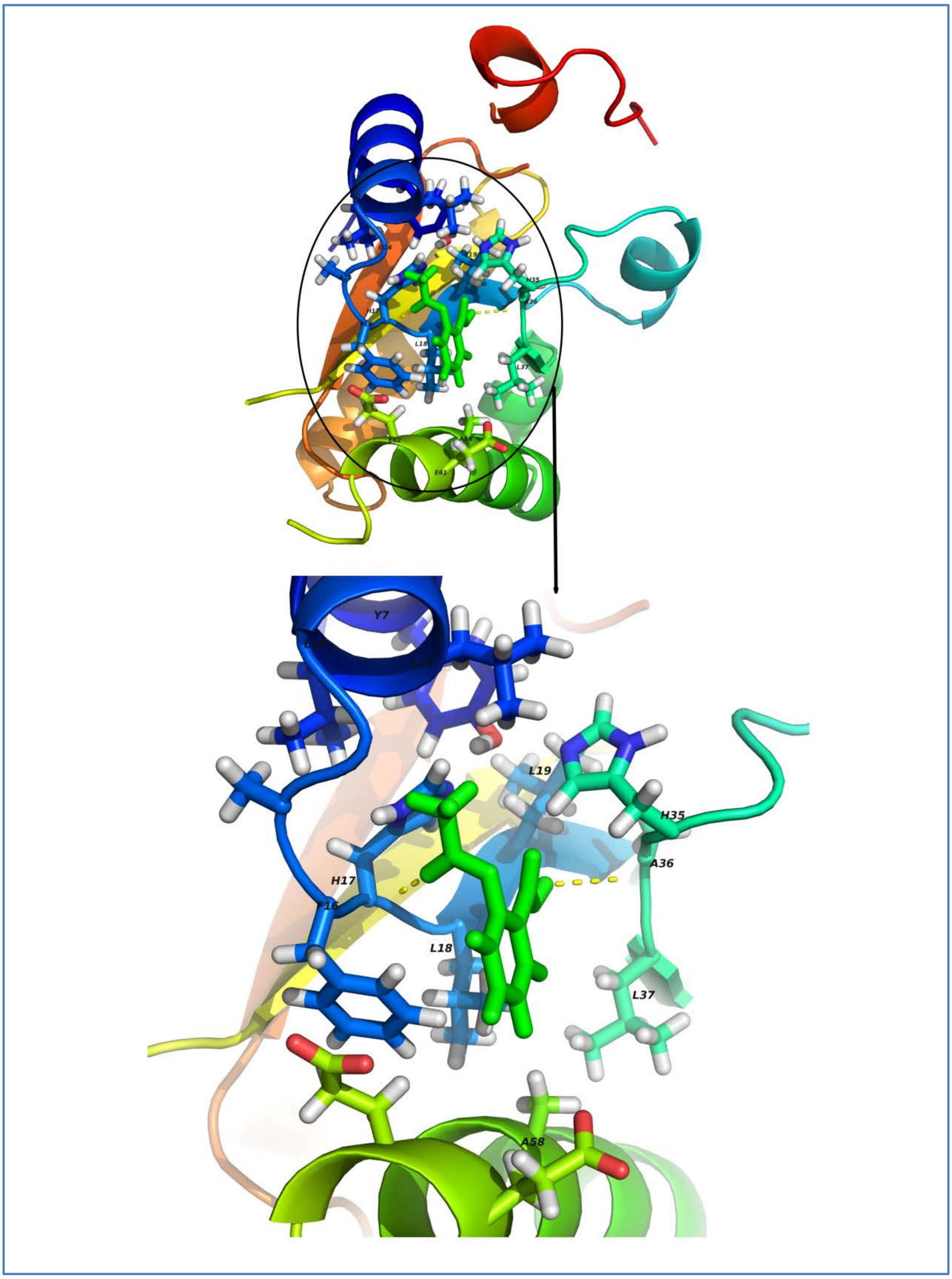

Figure S14. The binding mode of aspirin to the protein Adenylyltransferase and sulfurtransferase MOCS3 (MOCS3). The overview and close-up view of the binding mode of aspirin to MOCS3 were shown in the top and bottom panels, respectively. Secondary structural elements were colored from blue (N-terminus) to red (C-terminus). The compound aspirin is colored with green. The residues lying within 5 Ǻ from atoms of the aspirin are shown as sticks. The H-bonds are shown by dashed yellow line.

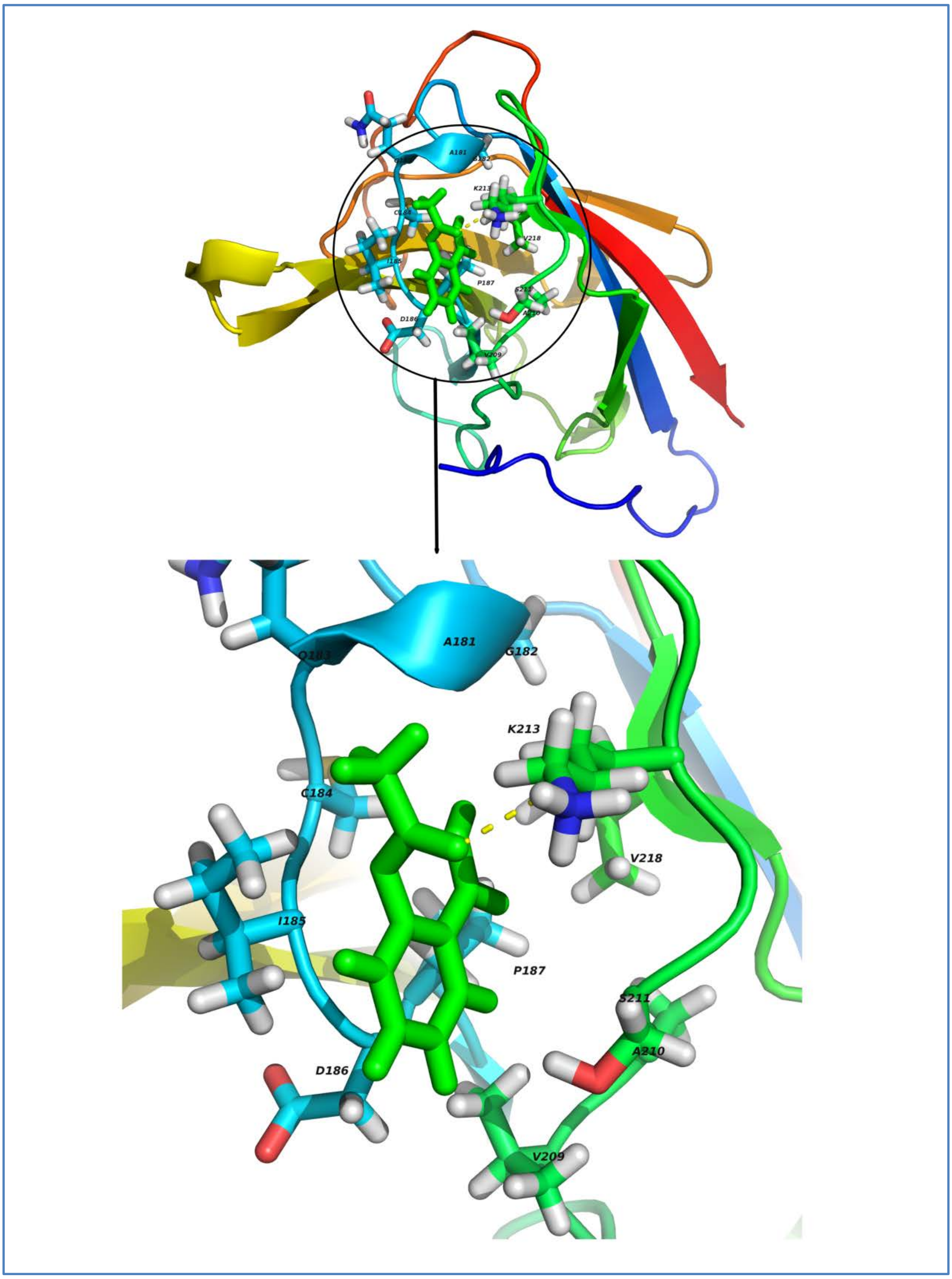

Figure S15. The binding mode of aspirin to the protein Axin interactor-dorsalization-associated protein (AIDA). The overview and close-up view of the binding mode of aspirin to AIDA were shown in the top and bottom panels, respectively. Secondary structural elements were colored from blue (N-terminus) to red (C-terminus). The compound aspirin is colored with green. The residues lying within 5 Ǻ from atoms of the aspirin are shown as sticks. The H-bonds are shown by dashed yellow line.

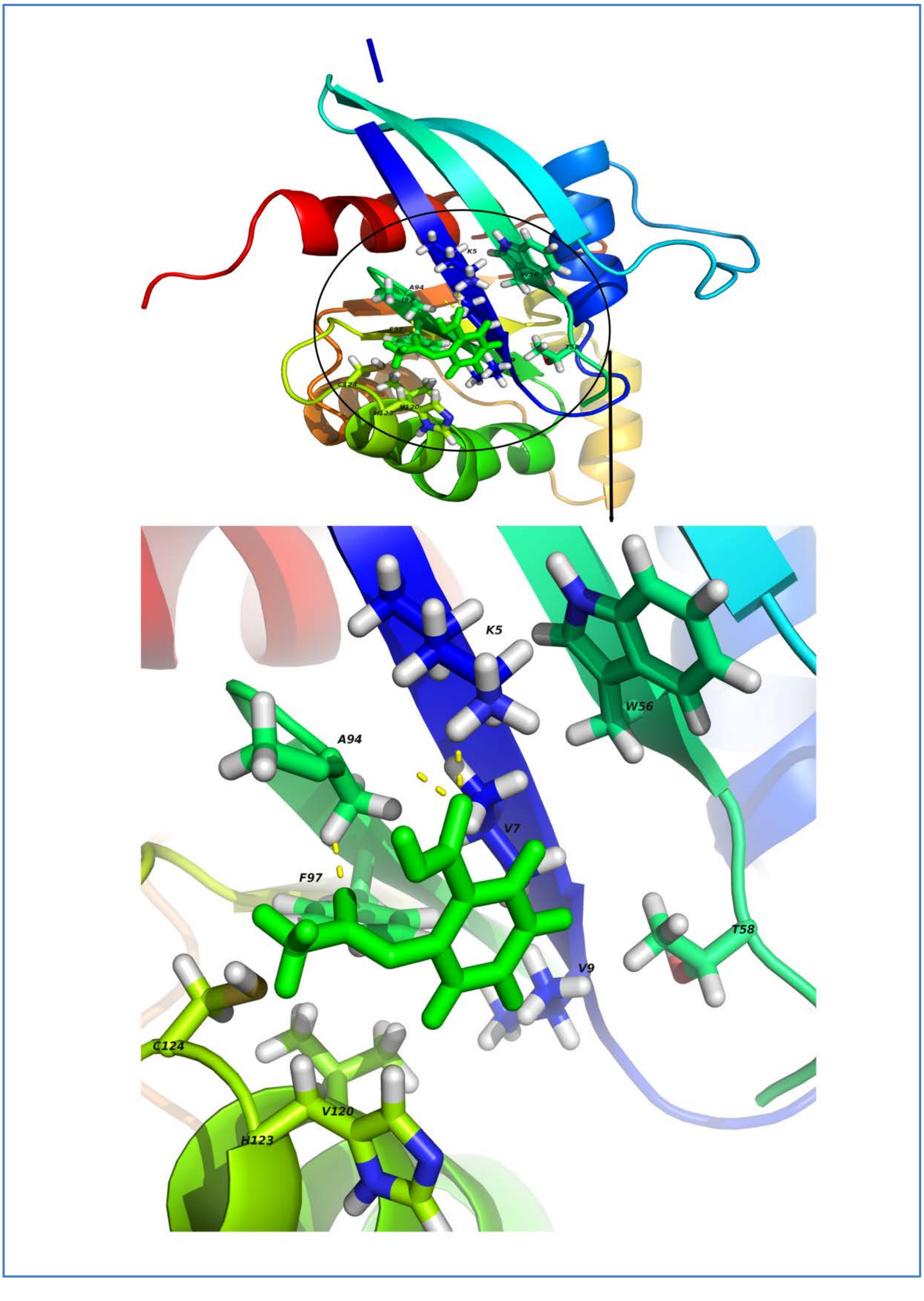

Figure S16. The binding mode of aspirin to the protein ras-related C3 botulinum toxin substrate 1 isoform Rac1b (RAC1). The overview and close-up view of the binding mode of aspirin to RAC1 were shown in the top and bottom panels, respectively. Secondary structural elements were colored from blue (N-terminus) to red (C-terminus). The compound aspirin is colored with green. The residues lying within 5 Ǻ from atoms of the aspirin are shown as sticks. The H-bonds are shown by dashed yellow line.

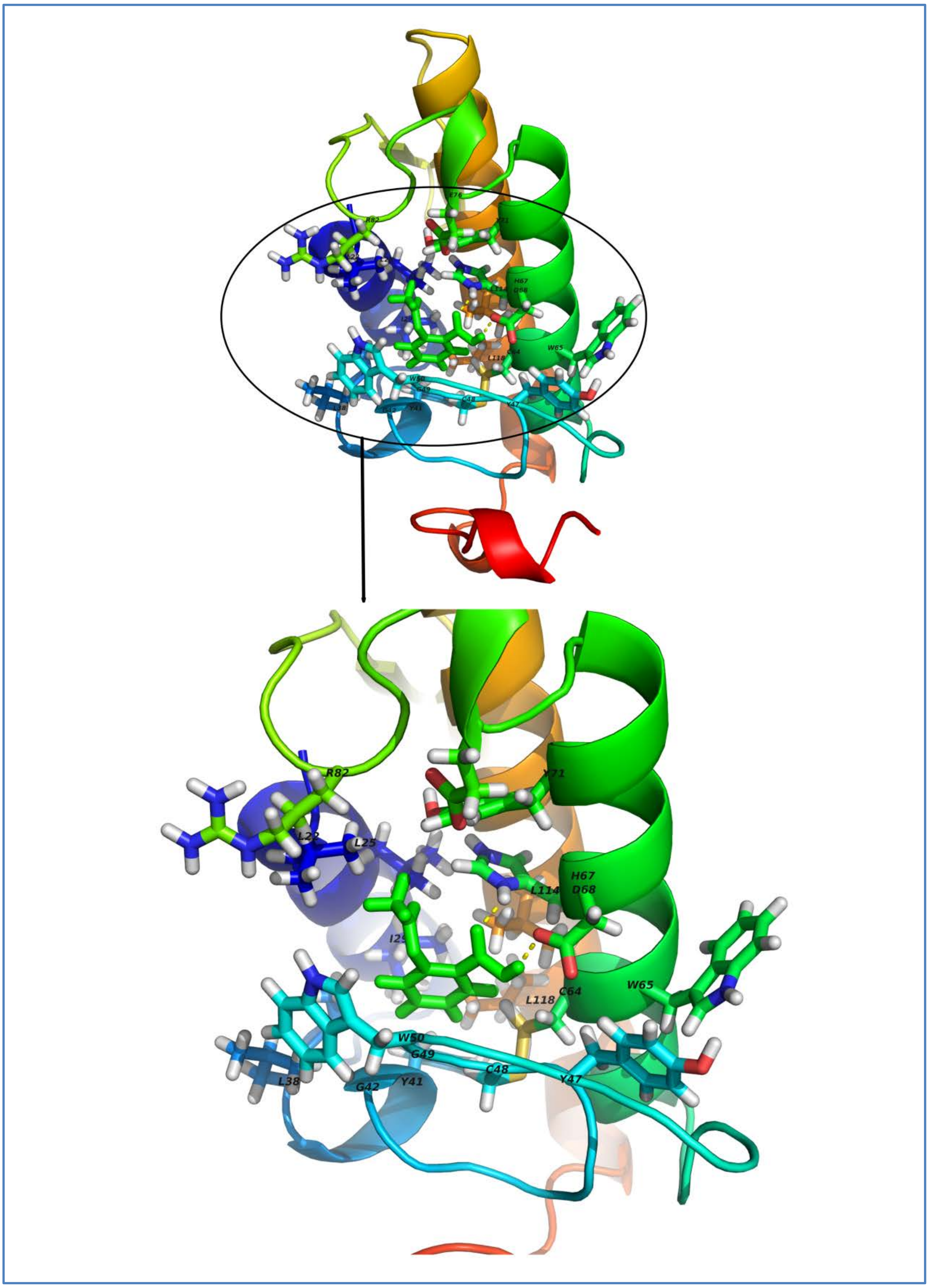

Figure S17. The binding mode of aspirin to the protein Calcium-dependent phospholipase A2 (PLA2G5). The overview and close-up view of the binding mode of aspirin to PLA2G5 were shown in the top and bottom panels, respectively. Secondary structural elements were colored from blue (N-terminus) to red (C-terminus). The compound aspirin is colored with green. The residues lying within 5 Ǻ from atoms of the aspirin are shown as sticks. The H-bonds are shown by dashed yellow line.

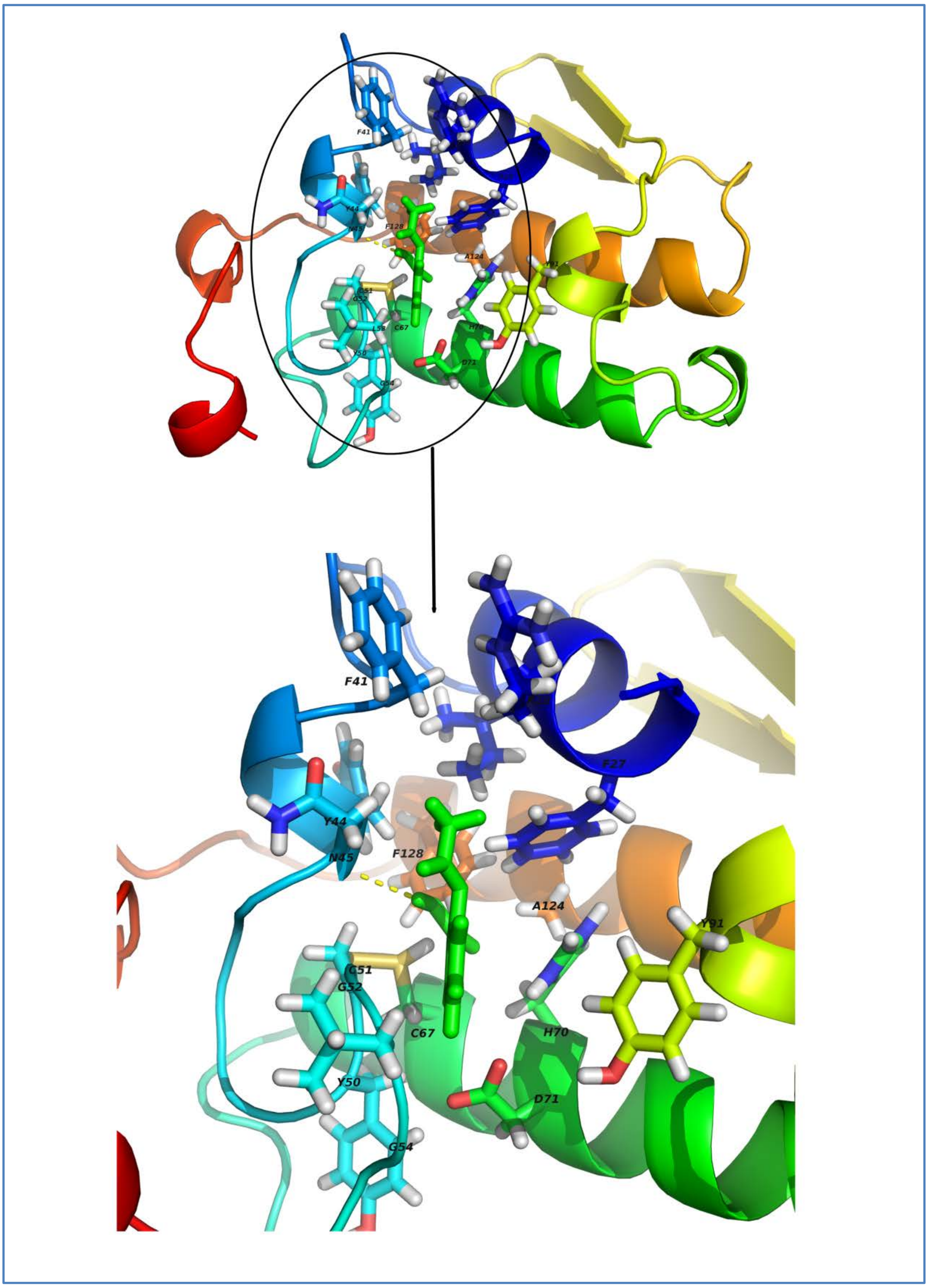

Figure S18. The binding mode of aspirin to the protein Phospholipase A2 (PLA2G1B). The overview and close-up view of the binding mode of aspirin to PLA2G1B were shown in the top and bottom panels, respectively. Secondary structural elements were colored from blue (N-terminus) to red (C-terminus). The compound aspirin is colored with green. The residues lying within 5 Ǻ from atoms of the aspirin are shown as sticks. The H-bonds are shown by dashed yellow line.

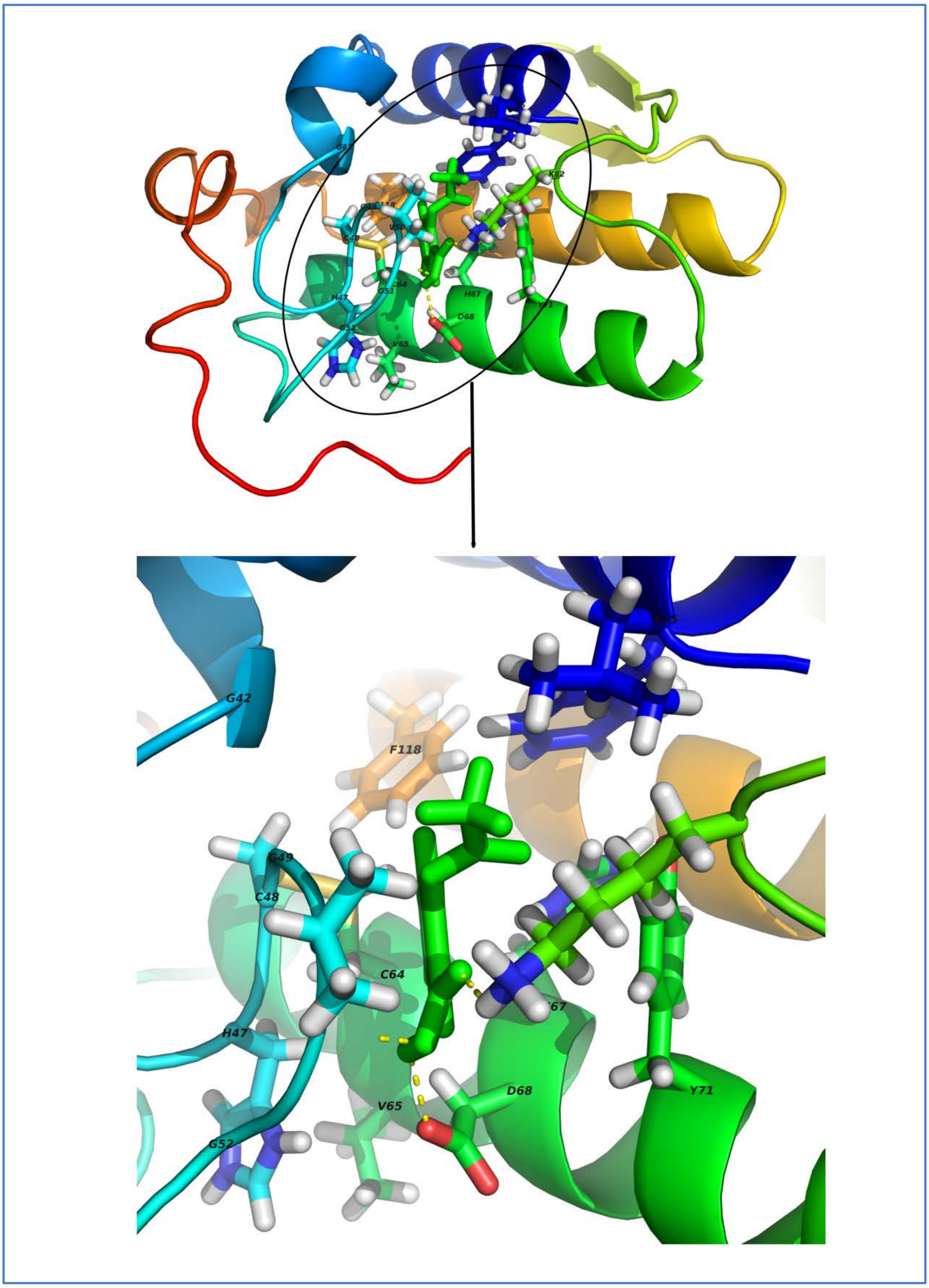

Figure S19. The binding mode of aspirin to the protein Phospholipase A2, membrane associated (PLA2G2A). The overview and close-up view of the binding mode of aspirin to PLA2G2A were shown in the top and bottom panels, respectively. Secondary structural elements were colored from blue (N-terminus) to red (C-terminus). The compound aspirin is colored with green. The residues lying within 5 Ǻ from atoms of the aspirin are shown as sticks. The H-bonds are shown by dashed yellow line.

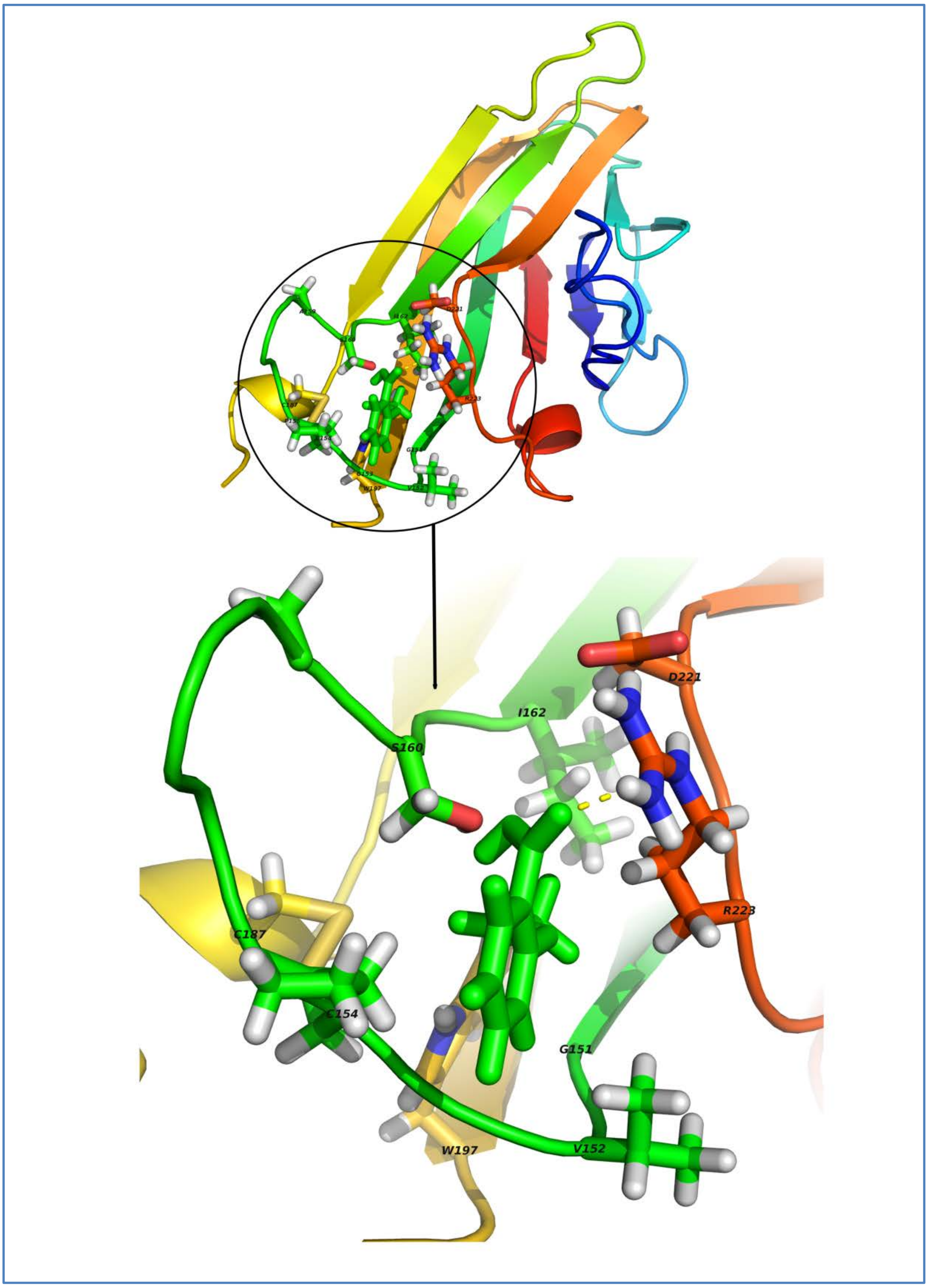

Figure S20. The binding mode of aspirin to the protein Tumor necrosis factor ligand superfamily member 14 (TNFSF14). The overview and close-up view of the binding mode of aspirin to TNFSF14 were shown in the top and bottom panels, respectively. Secondary structural elements were colored from blue (N-terminus) to red (C-terminus). The compound aspirin is colored with green. The residues lying within 5 Ǻ from atoms of the aspirin are shown as sticks. The H-bonds are shown by dashed yellow line.

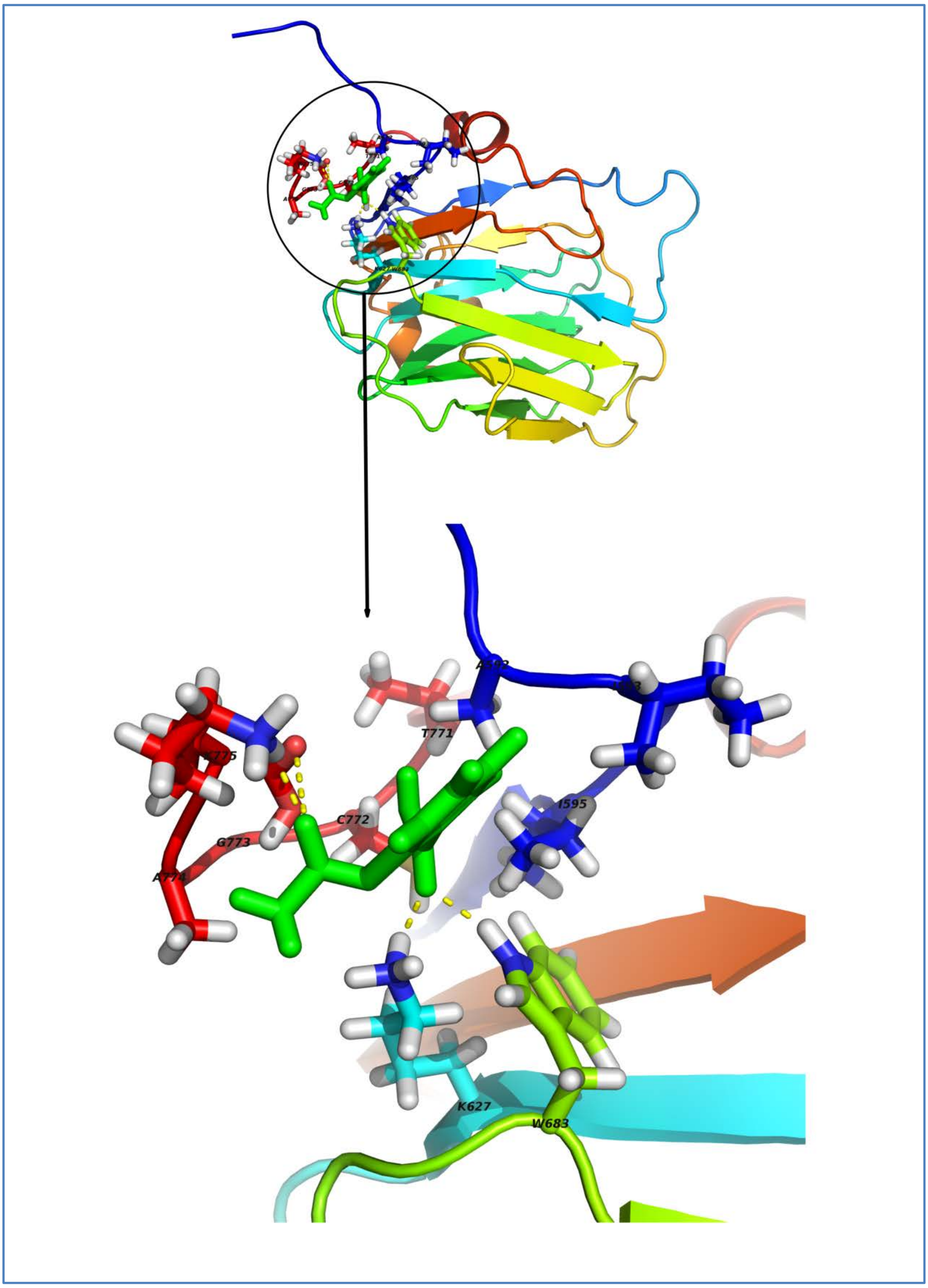

Figure S21. The binding mode of aspirin to the protein Pikachurin (EGFLAM). The overview and close-up view of the binding mode of aspirin to EGFLAM were shown in the top and bottom panels, respectively. Secondary structural elements were colored from blue (N-terminus) to red (C-terminus). The compound aspirin is colored with green. The residues lying within 5 Ǻ from atoms of the aspirin are shown as sticks. The H-bonds are shown by dashed yellow line.

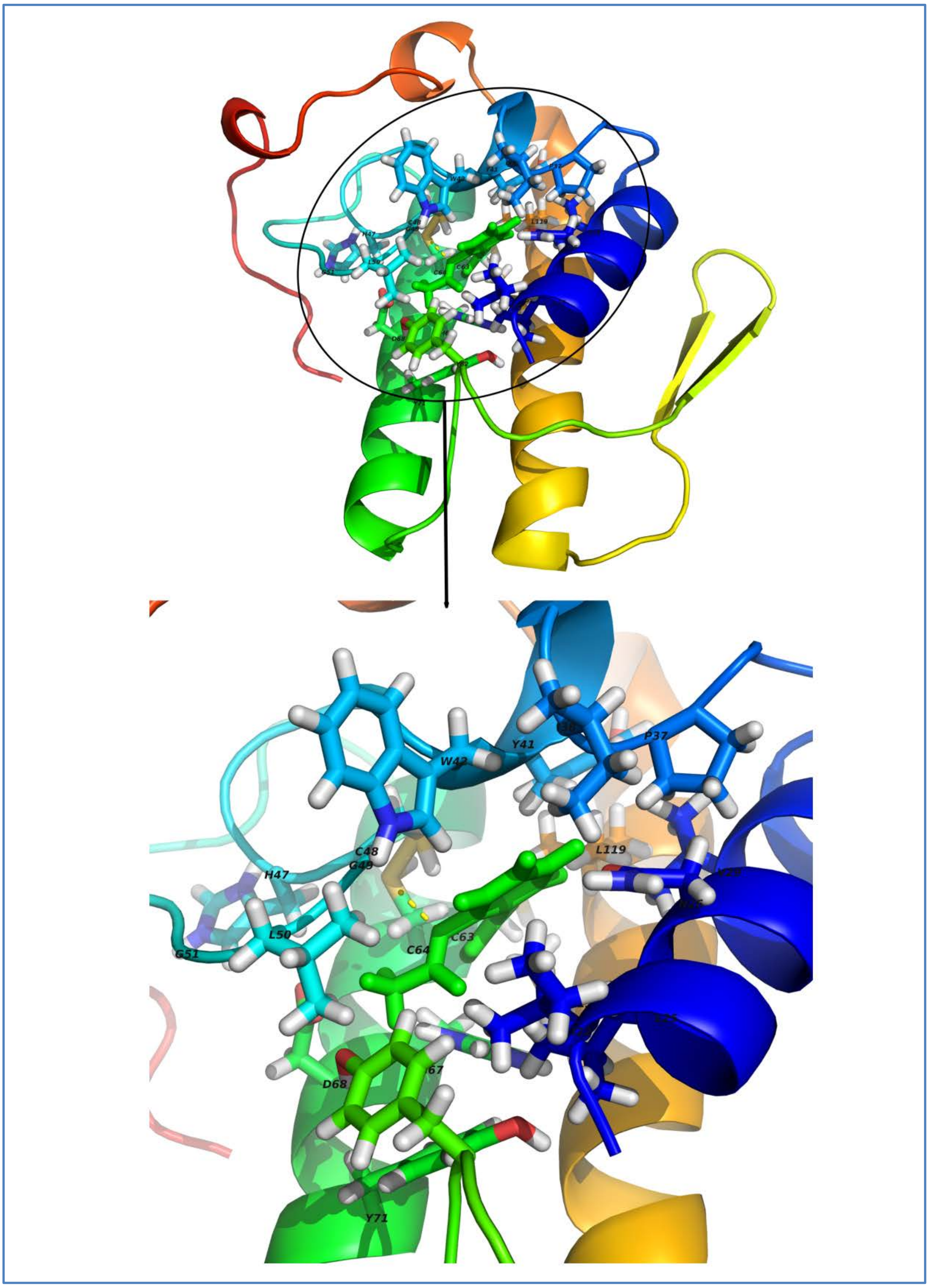

Figure S22. The binding mode of aspirin to the protein Group IID secretory phospholipase A2 (PLA2G2D). The overview and close-up view of the binding mode of aspirin to PLA2G2D were shown in the top and bottom panels, respectively. Secondary structural elements were colored from blue (N-terminus) to red (C-terminus). The compound aspirin is colored with green. The residues lying within 5 Ǻ from atoms of the aspirin are shown as sticks. The H-bonds are shown by dashed yellow line.

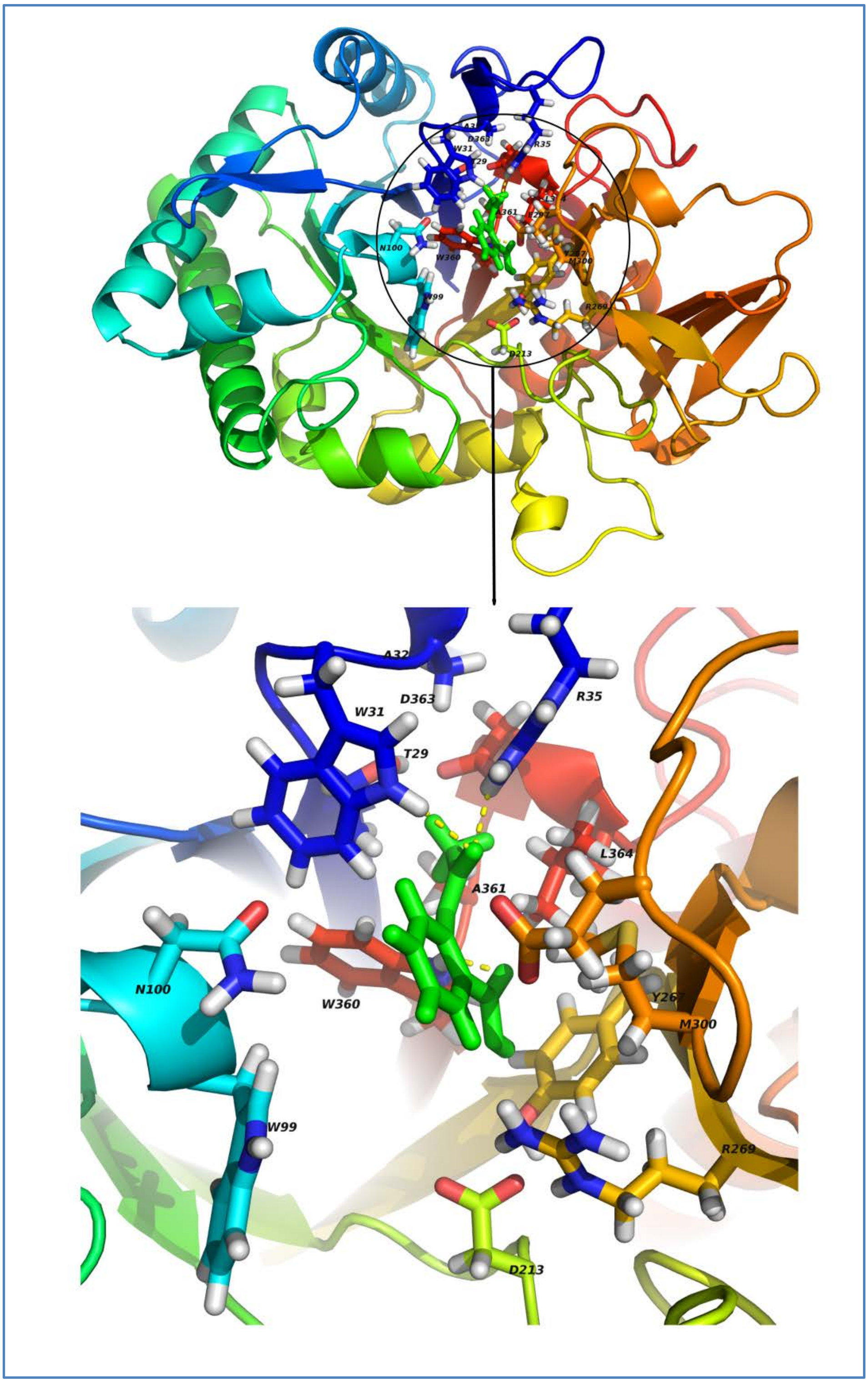

Figure S23. The binding mode of aspirin to the protein Chitotriosidase-1 (CHIT1). The overview and close-up view of the binding mode of aspirin to CHIT1 were shown in the top and bottom panels, respectively. Secondary structural elements were colored from blue (N-terminus) to red (C-terminus). The compound aspirin is colored with green. The residues lying within 5 Ǻ from atoms of the aspirin are shown as sticks. The H-bonds are shown by dashed yellow line.

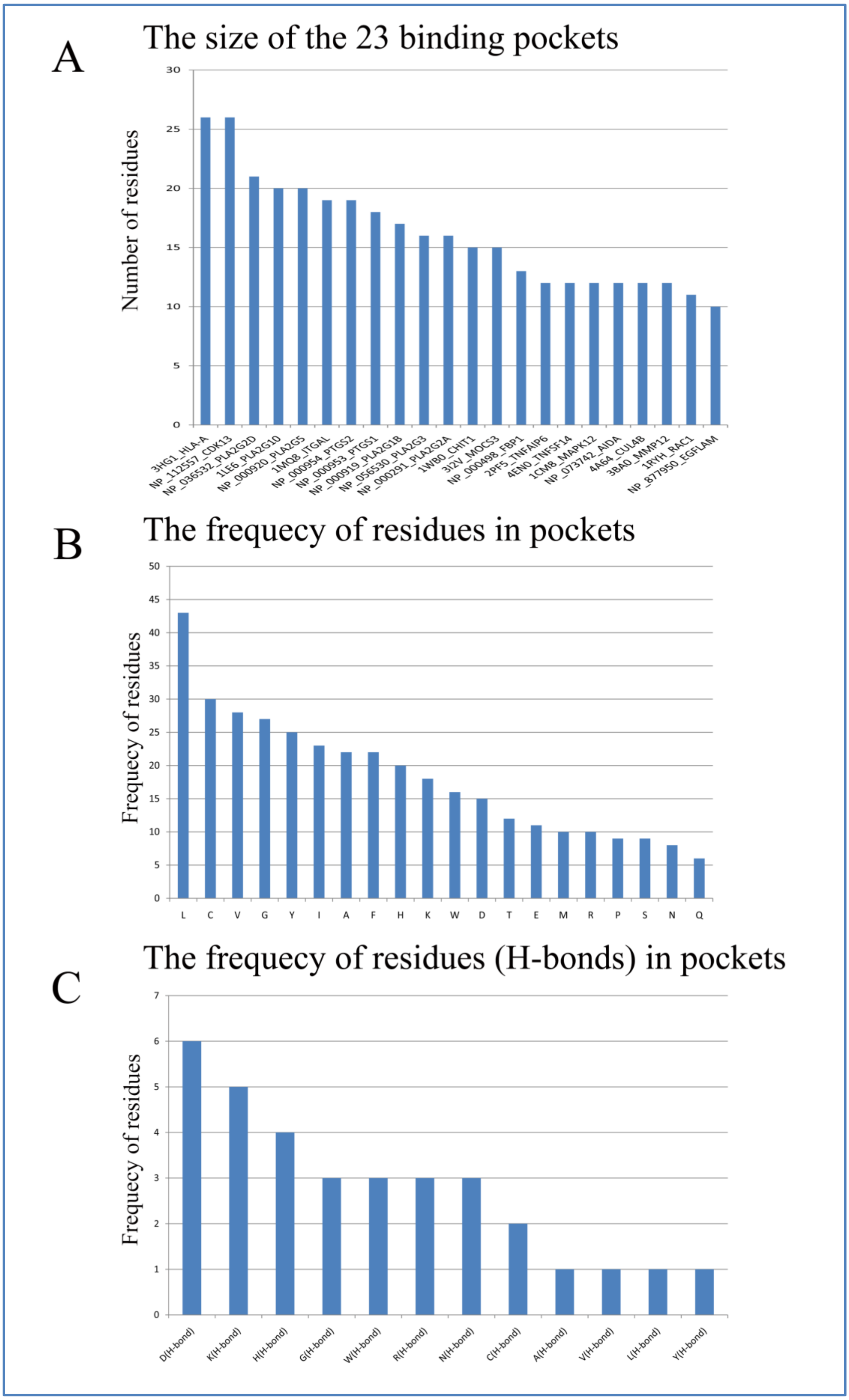

Figure S24. The statistic of 23 putative binding pockets of aspirin. **A)** The size of the 22 binding pockets of aspirin. **B)** The frequency of residues in all binding pockets of aspirin. **C)** The frequency of residues involved in formation of H-bond in all binding pockets of aspirin.
